# Diversity and Functionality of the Gut Microbiota in Hypertensive Patients from Different Regions of Peru

**DOI:** 10.12688/f1000research.170893.2

**Published:** 2026-09-28

**Authors:** Pool Marcos-Carbajal, Milagros Nicole Villegas-Suarez, Vasty Vanessa Rafael Ccama, Miguel Angel Otiniano Trujillo, Salomon Huancahuire-Vega, Mario Salomon Chambi Quispe, Alvaro Luque Arapa, Manuel Ruiz Panaifo, Pilar Elizabeth Diaz Rengifo, Cinthia Esther Sias Garay, Ruben Espinoza Rojas, Raul Tito, Sonia Rohra Benitez, Silvana Teresa Tapia Paniagua

**Affiliations:** 1 Laboratorio de Investigación en Biología Molecular, Universidad Peruana Unión, Lurigancho-Chosica, Peru; 2 Facultad de Medicina Humana, Universidad Peruana de Ciencias Aplicadas, Chorrillos, Peru; 3 Clínica Americana Juliaca, Juliaca, Peru; 4 Clínica Adventista Ana Stahl, Iquitos, Peru; 5 Laboratorio de Referencia Regional Salud Publica San Martin, Laboratorio de Referencia Regional Salud Publica San Martin, Tarapoto, San Martin, Peru; 6 Language Center, Peruvian Union University, Lurigancho-Chosica, Peru; 7 Instituto de Investigacion en Ciencias Biomédicas, Universidad Ricardo Palma, Santiago de Surco, Peru; 8 Department of Microbiology and Immunology, Rega Institute for Medical Research, Katholieke Universiteit Leuven Rega Institute for Medical Research, Leuven, Flanders, Belgium; 9 Department of Microbiology, University of Malaga, Malaga, Andalusca, Spain

**Keywords:** High blood pressure, gut microbiota, microbiome diversity, Peru

## Abstract

**Introduction:**

Hypertension affects approximately 14.2% of people over 15 years of age in Peru, constituting a major public health issue. Recent evidence highlights the gut microbiome’s role in regulating blood pressure through bioactive metabolites that influence inflammation and metabolism. This study aims to characterize the gut microbiota of hypertensive adults from three Peruvian regions, contributing to the understanding of microbial influences on hypertension.

**Objective:**

To analyze the composition and functionality of the gut microbiota in hypertensive and normotensive adults from different Peruvian regions, considering age, body mass index (BMI), and geographical location.

**Methods:**

An observational, cross-sectional, and analytical study was conducted with 84 adults (46 hypertensive and 38 normotensive) from nine regions of Peru. Fecal samples were collected for 16S rRNA gene amplicon sequencing of the V3–V4 region. Alpha and beta diversity were evaluated using the Shannon index, UniFrac distances, and multivariate analyses. Microbial functional pathways were examined to identify potential metabolic differences between groups.

**Results:**

The dominant phyla in all groups were Bacillota and Bacteroidota. At the genus level, *Streptococcus* was associated with hypertensive patients with BMI ≥25, while *Alistipes* appeared only in normotensive individuals with BMI <25. Alpha diversity was greater in hypertensive participants under 50 years and with BMI <25, while lower diversity was seen in normotensive adults over 50 and with BMI ≥25. Beta diversity analyses showed substantial overlap across hypertensive status, age, and regional groups, with no clear separation. Adjusted multivariable models confirmed no significant independent association between hypertension status and either alpha or beta diversity after accounting for age, BMI, and region, with region remaining the strongest independent correlate of community composition (R ^2^ = 16.3%, p = 0.001). Functional profiles were similar among groups, mainly involving carbohydrate, amino acid, and lipid metabolism, though BMI and age modulated these patterns.

**Conclusions:**

Gut microbiota composition and diversity in this Peruvian cohort were associated not only with hypertension status but also with anthropometric and geographic factors. Despite similar functional profiles between groups, environmental and nutritional context appeared to have a greater influence than hypertension status alone. These exploratory, hypothesis-generating findings should be confirmed in larger, adjusted, longitudinal studies before informing preventive or therapeutic strategies for local settings.

## Introduction

Hypertension is one of the main public health problems due to its increasing morbidity and mortality. It affects one in three adults worldwide, totaling more than 1.28 billion people. Most of them live in low or middle-income countries, of whom only 42% have their condition under control. ^1^ According to the Pan American Health Organization (PAHO), ^2^ it is defined as a systolic blood pressure equal to or greater than 140 mmHg or a diastolic blood pressure equal to or greater than 90 mmHg, and is one of the most important risk factors for cardiovascular disease, chronic kidney disease, and stroke. In Peru, according to a report by the National Institute of Statistics and Informatics (INEI) 2024, ^3^ hypertension was detected in 14.2% of the population over 15 years of age, as measured by the ENDES (Demographic and Family Health Survey). The male population is the most affected (18.3%) compared to the female population (10.3%). At the regional level, the coast had the highest percentage (15.8%), with a lower prevalence in the highlands (12.1%) and the jungle (9.8%).

Although the role of the gut microbiome in blood pressure regulation is increasingly demonstrated and recognized, most studies have been conducted in high-income countries, where dietary and environmental exposures differ considerably from those in Latin America. Therefore, evidence from low- and middle-income countries remains scarce, and data from Peru are virtually nonexistent. This knowledge gap limits our understanding of how regional factors such as altitude, traditional diets, and sociocultural diversity may influence interactions between the microbiome and hypertension.

Meanwhile, the gut microbiota, an ecosystem of microorganisms, has been recognized as an important factor in cardiovascular health. Several studies have identified that alterations in its composition, known as dysbiosis, may play a significant role in the development and progression of hypertension. ^4^ The interaction between the microbiome and blood pressure occurs through multiple mechanisms, such as the production of short-chain fatty acids (SCFAs), which contribute to the regulation of inflammation and vascular homeostasis. ^5– 7^ For example, propionate and butyrate, metabolites produced by gut bacteria, have anti-inflammatory and antihypertensive properties that have been documented in animal and human models. ^8^

Furthermore, dysbiosis can promote systemic inflammation by translocating bacterial products such as lipopolysaccharides into the systemic circulation, activating immune responses that contribute to endothelial dysfunction and, consequently, increased blood pressure. ^9, 10^ Recent studies have shown that hypertensive patients have reduced microbial diversity and elevated levels of inflammatory bacteria, highlighting the relevance of this gut-heart axis in the pathophysiology of hypertension. ^11^

Based on previous findings, we hypothesized that hypertensive patients would exhibit reduced microbial diversity and distinct taxonomic shifts compared to normotensive individuals. Moreover, we anticipated that these differences might vary according to age, BMI, and geographical location, reflecting the heterogeneous environmental and nutritional contexts across Peru.

To test this hypothesis, we conducted a cross-sectional study in nine Peruvian regions, aiming to characterize the gut microbiota composition of hypertensive and normotensive adults, to evaluate its association with demographic and anthropometric factors, and to explore regional patterns that may reflect environmental and nutritional diversity. The findings of this study have potential implications at multiple levels: regionally, to inform strategies for the prevention and management of hypertension in high-altitude populations; nationally, to support health policies tailored to Peru’s geographic and cultural diversity; and globally, to expand the understanding of microbiome–hypertension interactions and to guide the development of context-specific therapeutic interventions

## Material and methods

### Design and population

The study design is observational, cross-sectional, and analytical. The study population consisted of adults who participated in health campaigns conducted in nine departments of Peru (Coastal: La Libertad, Lambayeque, Lima; Highlands: Arequipa, Cusco, Puno; and Jungle: Ucayali, Loreto, San Martín). These regions were selected for convenience, as they have trained health professionals who will facilitate data and sample collection and provide support with campaign logistics, ensuring the proper development of the study.

### Inclusion and exclusion criteria

Participants were selected non-probabilistically for convenience. The following inclusion criteria were used: a) Male or female subjects aged 18 years or older, with no upper age limit set a priori; b) Subjects must be of Peruvian or other nationality but must have resided for at least six months in the region where the participant will be assessed; c) Subjects willing and able to provide blood for blood count and lipid profile testing, as well as a stool sample; d) Must be able to provide signed and dated informed consent.

The following exclusion criteria were used: a) Participants with secondary arterial hypertension b) Recent use of antibiotics or probiotics in the last 3 months c) Pregnant or breastfeeding women d) People with recent major surgery or trauma, endocrine diseases ( *Diabetes mellitus*, hypo/hyperthyroidism), autoimmune diseases (lupus, psoriasis, collagen diseases), cancer of any etiology and significant respiratory/cardiac alteration (CHF, COPD, pneumonia, asthma and bronchitis).

### Procedures

1. Clinical research record

Information was collected through patient clinical records on age, sex, height, weight, diet, blood pressure (systolic and diastolic), and oxygen saturation. A stool sample was also requested from patients treated during health campaigns conducted in Coastal (Lima, Chiclayo, Trujillo); Highlands (Arequipa, Cusco, Puno) and Jungle (Ucayali, San Martín, and Loreto). The information obtained was systematized in a Microsoft Excel database, ensuring correct entry for subsequent analysis.

2. Clinical analysis

The previously systematized database was imported into STATA SE version 18 software, where patients who met the diagnostic criteria for hypertension were identified and categorized. According to the World Health Organization, ^1^ hypertension is defined as a systolic blood pressure ≥140 mmHg and/or diastolic blood pressure ≥90 mmHg. This in two or more measurements under suitable conditions.

3. Gut microbiome analysis

The availability of participants’ stool samples was verified. After completing the survey, participants were given a stool collection tube, labeled with their name and region, as the sample would be collected by the patient themselves at home. Participants were asked to submit their stool sample the following day, on an empty stomach, and as recently as possible. Survey data was collected to account for any pathologies or medication the participant had. Once identified, all samples were transported to the Molecular Biology Research Laboratory (LIBM) at the Universidad Peruana Unión (UPeU) under surveillance, thus ensuring their integrity. The stool samples were classified according to their storage method. One group was stored at -20°C, while the other was kept at room temperature for later analysis, which was used for the analysis of parasitic infections. This storage protocol was applied consistently to all participants across all nine regions, so differences in sample handling are unlikely to account for the regional differences in microbiota composition reported below.

### DNA extraction and quantification

Total DNA extraction was performed in duplicate using the EasyPure Stool Genomic DNA Kit, following the manufacturer’s recommendations. The extracted DNA was quantified using the Qubit 1X dsDNA BR Assays Kit, and only the sample with the highest DNA concentration was used for subsequent molecular analyses.

### 16S ARNr gene sequencing and bioinformatics analysis

The V3–V4 hypervariable region of the 16S rRNA gene was amplified with primers 341F and 806R and sequenced using the Illumina MiSeq platform (2 × 250 bp paired-end; Novogene Europe, Cambridge, UK). Raw reads were trimmed to remove primers with *cutadapt*, and quality was assessed with *FastQC.* ^12^ Sequence processing was performed in R using the *DADA2* pipeline (Callahan et al., 2016), including quality filtering, error modeling, denoising, merging of paired reads, and chimera removal. Amplicon sequence variants (ASVs) were assigned taxonomically against the SILVA database (release 138) clustered at 99% identity.

ASV abundance tables were analyzed in R with the *phyloseq* ^14^ and *vegan* ^15^ packages. Alpha diversity (Shannon index) was calculated to estimate richness and evenness, while beta diversity was visualized by non-metric multidimensional scaling (NMDS) using UniFrac distances. Group differences in diversity were tested with one-way ANOVA ( *p* < 0.05). ASVs with fewer than 10 reads in at least 10% of samples were excluded from downstream analyses.

Taxonomic composition was visualized using *ggplot2*, and differential abundance analysis was performed with *DESeq2,* ^13^ applying a false discovery rate (FDR) threshold of <0.05. Functional predictions of microbial communities were inferred with *PICRUSt2.*

### Statistical analysis

The analysis plan followed the following phases: (1) Data preparation and classification, where information from the research clinical records was systematized in a database using Microsoft Excel. An initial data verification was performed to identify inconsistencies or data entry errors. The systolic blood pressure and diastolic blood pressure variables, initially measured as numerical values, were reclassified using STATA SE version 18 software into a binary categorical variable: With hypertension: Systolic blood pressure values ≥140 mmHg or diastolic blood pressure ≥90 mmHg. Without hypertension: Systolic blood pressure values <140 mmHg and diastolic blood pressure <90 mmHg. The new classification was documented in the database as a categorical variable for analysis. (2) Univariate analysis, in which each of the variables was described according to its nature (categorical with absolute and relative frequencies, and numerical with measures of central tendency and dispersion). In addition, to determine whether the numerical variables follow a normal distribution, statistical tests such as the Shapiro-Wilk test were applied. (3) Bivariate analysis, where the associations between hypertension and the other categorical variables were compared using the chi-square test. While to compare hypertension with the numerical variables, the Student t test for independent samples was applied (if the data have a normal distribution), or the Mann-Whitney U test (if the data do not follow a normal distribution). (4) Adjusted multivariable analysis. To evaluate whether hypertension status was independently associated with microbiota composition after accounting for region, age, and BMI, the ASV-level relative-abundance table (83 of 84 samples passing sequencing quality control; one sample was not sequenced) was used to compute Bray-Curtis dissimilarities between samples. A phylogenetic tree was not available for this analysis, so UniFrac distances could not be used here; Bray-Curtis dissimilarity was used instead as a standard alternative for compositional data. Region, age group (<50 vs. ≥50 years), BMI group (<25 vs. ≥25), and hypertension status were entered sequentially into a permutational multivariate analysis of variance (PERMANOVA; 999 permutations), with hypertension entered last so that its association could be evaluated after accounting for the other three factors. Shannon alpha diversity was similarly modeled using multiple linear regression with the same four covariates, and Type II sums of squares were used to obtain the covariate-adjusted significance of each term.

## Results

### Clinical characteristics of the study participants

A total of 84 participants were included, of whom 46 were hypertensive (H1) and 38 normotensive (H0). Clinical characteristics were comparable between groups, except for higher mean blood pressure values in the hypertensive group ( Table 1).

**Table 1.** Bivariate analysis of factors associated with the gut microbiota of hypertensive patients. Qualitative variables were tested using the Chi-square test with Yates correction, and quantitative variables were tested using the Mann-Whitney U test.

Variables		Hypertensive						P value
		Total		No		Yes		
		N	%	N	%	N	%	
**Gender**	Total	84	100,0%	38	45,2%	46	54,8%	0.470
	Female	45	100,0%	22	48,9%	23	51,1%	
	Male	39	100,0%	16	41,0%	23	59,0%	
**Parasitological Results**	Total	84	100,0%	38	45,2%	46	54,8%	0.199
	Negative	73	100,0%	35	47,9%	38	52,1%	
	Positive	11	100,0%	3	27,3%	8	72,7%	
**Region**	Total	84	100,0%	38	45,2%	46	54,8%	0.733
	Highlands	12	100,0%	6	50,0%	6	50,0%	
	Coast	44	100,0%	21	47,7%	23	52,3%	
	Jungle	28	100,0%	11	39,3%	17	60,7%	
**Age**		54.5+/-13.7		47.4+/-12.3		60.4+/-11.9		<0.001
**Systolic blood pressure (mmHg)**		127.7+/-18.7		113.9+/-11.6		139.4+/-15.3		<0.001
**Diastolic blood pressure (mmHg)**		81.4+/-9		78.9+/-9.6		83.5+/-8.1		<0.05
**Weight (Kg)**		71.9+/-15.5		74.4+/-16.2		69.9+/-14.8		0.186
**Height (cm)**		1.6+/-0.1		1.7+/-0.2		1.6+/-0.1		0.160
**Body mass index (Kg/m** ^**2**^ **)**		28.3+/-5.6		28.8+/-6.3		27.8+/-5		0.416
**Red blood cells (cel/mm** ^**3**^ **)**		4571428.6+/-484726.1		4598421.1+/-487179.1		4549130.5+/-486926.1		0.646
**Hemoglobin (g/dL)**		14+/-1.8		14+/-1.8		14+/-1.9		0.877
**Hematocrit**		41.6+/-4.8		41.4+/-4.8		41.6+/-4.9		0.839
**White blood cells (cel/mm** ^**3**^ **)**		7714+/-7122.2		7150.8+/-1454.3		8179.2+/-9556.7		0.513
**Platelets (mcl)**		267972.2+/-99035		288467.7+/-119142.7		251041.1+/-75967.1		0.085
**Total Cholesterol (mg/dL)**		186.8+/-37.5		179.9+/-35.1		192.5+/-38.7		0.125
**HDL-Cholesterol**		49.1+/-11.2		47.2+/-10.2		50.7+/-11.9		0.158
**LDL-Cholesterol**		104.8+/-34.7		100.6+/-32.5		108.3+/-36.5		0.314
**VLDL-Cholesterol**		31+/-13.7		33.3+/-15.7		29.1+/-11.7		0.164
**Triglycerides (mg/dL)**		171.5+/-91.7		179.9+/-92.6		164.5+/-91.4		0.446
**Glucose (mg/dL)**		124.9+/-73.9		139.5+/-98		112.8+/-42.9		0.099

Among the 46 hypertensive patients included in Table S1, the distribution by sex was nearly balanced, with 50% females and 50% males overall, although regional variation was evident (e.g., Cusco included only male patients, while Chiclayo showed a predominance of males as well). Parasitological analysis revealed that 17.4% of hypertensives were positive for intestinal parasites; all positive samples across the cohort (11/84) were identified as Entamoeba coli cysts, a protozoan generally regarded as a non-pathogenic intestinal commensal rather than a disease-causing parasite, with higher proportions in Chiclayo and Loreto. Geographically, most hypertensive patients came from coastal regions (50%), followed by the jungle (37%) and the highlands (13%). Regarding pharmacological management, 45 of the 46 hypertensive participants (97.8%) were receiving antihypertensive treatment at the time of enrollment; most were on an ACE inhibitor (captopril or enalapril, 34/46, 73.9%) or an angiotensin receptor blocker (losartan, alone or combined with amlodipine, 10/46, 21.7%), and only one participant reported taking no medication. Mean age was 60.4 ± 11.9 years, with older individuals particularly represented in Puno/Juliaca and Trujillo. Systolic and diastolic blood pressure values varied by site, but the overall mean remained above the diagnostic threshold for hypertension. Other clinical parameters such as BMI, hematological indices, and lipid profiles showed regional variability, suggesting heterogeneity in the metabolic status of hypertensive patients across Peru ( Table 2).

**Table 2.** Clinical and demographic characteristics of hypertensive patients stratified by Peruvian regions. Distribution of sex, parasitological results, geographic location, and selected clinical parameters among 46 hypertensive participants from nine regions of Peru. (a): Chi-square test with Yates correction, SD: Standard Deviation, NA: Not applicable, because there are no values in certain categories, small sample, IQR: Interquartile Range.

Variables		Regions																				
		Total		Arequipa		Chiclayo		Cuzco		Lima		Loreto		Pucallpa		Puno/Juliaca		Tarapoto		Trujillo		
		n	%	n	%	n	%	n	%	n	%	n	%	n	%	n	%	n	%	n	%	
**Gender**	Total	46	100.00%	4	100.00%	5	100.00%	2	100.00%	11	100.00%	8	100.00%	5	100.00%	2	100.00%	4	100.00%	5	100.00%	0.578
	Female	23	50.00%	2	50.00%	1	20.00%	0	0.00%	7	63.60%	4	50.00%	2	40.00%	1	50.00%	2	50.00%	4	80.00%	
	Male	23	50.00%	2	50.00%	4	80.00%	2	100.00%	4	36.40%	4	50.00%	3	60.00%	1	50.00%	2	50.00%	1	20.00%	
**Parasitological Results**	Total	46	100.00%	4	100.00%	5	100.00%	2	100.00%	11	100.00%	8	100.00%	5	100.00%	2	100.00%	4	100.00%	5	100.00%	0.807
	Negative	38	82.60%	4	100.00%	3	60.00%	2	100.00%	10	90.90%	6	75.00%	4	80.00%	2	100.00%	3	75.00%	4	80.00%	
	Positive	8	17.40%	0	0.00%	2	40.00%	0	0.00%	1	9.10%	2	25.00%	1	20.00%	0	0.00%	1	25.00%	1	20.00%	
**Region**	Total	46	100.00%	4	100.00%	5	100.00%	2	100.00%	11	100.00%	8	100.00%	5	100.00%	2	100.00%	4	100.00%	5	100.00%	0
	Highlands	6	13.00%	4	100.00%	0	0.00%	2	100.00%	0	0.00%	0	0.00%	0	0.00%	0	0.00%	0	0.00%	0	0.00%	
	Cost	23	50.00%	0	0.00%	5	100.00%	0	0.00%	11	100.00%	0	0.00%	0	0.00%	2	100.00%	0	0.00%	5	100.00%	
	Jungle	17	37.00%	0	0.00%	0	0.00%	0	0.00%	0	0.00%	8	100.00%	5	100.00%	0	0.00%	4	100.00%	0	0.00%	
**Age (Mean +/- SD)**		60.4+/- 11.9		63.3+/- 12		52.6+/- 16.8		55.5+/- 3.6		59.5+/- 10.2		60+/- 12.7		52.4+/- 10.1		78+/- 4.3		61.5+/- 5.6		70.4+/- 7.3		0.088
**Blood Pressure (Median +/- IQR)**		139.4+/- 15.3		154.5+/- 24.6		130.8+/- 8.5		122.5+/- 17.7		136.4+/- 14.2		140.5+/- 13.4		132.2+/- 10.8		149.5+/- 22		144+/- 7.9		148+/- 13.1		0.122
**Blood Pressure (Mean +/- SD)**		83.5+/- 8.1		90+/- 6		84.4+/- 3.9		85+/- 21.3		78.5+/- 9.9		86.2+/- 6.7		84.6+/- 2.8		85.5+/- 13.5		79.7+/- 4.6		84+/- 5.5		0.361
**Weight (Mean +/- SD)**		69.9+/- 14.8		75.5+/- 11.2		49+/- 4.7		80.5+/- 16.3		73.8+/- 12.9		72.7+/- 17.1		79.6+/- 14.2		75+/- 7.1		58.5+/- 7.3		66+/- 11.2		0.01
**Height (Mean +/- SD)**		1.6+/- 0.1		1.7+/- 0.1		1.6+/- 0.1		1.7+/- 0.1		1.6+/- 0.1		1.6+/- 0.1		1.7+/- 0.1		1.6+/- 0.2		1.7+/- 0.1		1.6+/- 0.1		0.441
**Body Mass Index (Mean +/- SD)**		27.8+/- 5		28.2+/- 3.6		20.8+/- 1.5		29+/- 4.2		29.6+/- 4.4		29.8+/- 4.3		30.4+/- 4.2		32.2+/- 7.7		22.5+/- 0.7		26.9+/- 4.4		0.002
**Red Blood Cells (Mean +/- SD)**		4549130.5+/- 486926.1		4975000+/- 492442.9		4672000+/- 175128.6		5405000+/- 247487.4		4468181.9+/- 331868		4133750+/- 182751.8		4722000+/- 757344.1		4535000+/- 1067731.3		4712500+/- 255522.4		4288000+/- 345210.1		0.008
**Hemoglobin (Median +/- IQR)**		14+/- 1.9		14.4+/- 1.5		14.3+/- 0.5		18.9+/- 0.4		13.3+/- 1.3		13.7+/- 1.4		14.1+/- 1.7		15.5+/- 4.7		13.6+/- 1.2		12.7+/- 1.2		0.001
**HTN (Mean +/- SD)**		41.6+/- 4.9		43.5+/- 4.6		41.8+/- 1.2		52.7+/- 1.9		40.6+/- 3.9		41.5+/- 4.3		41.8+/- 5.3		43.1+/- 12.1		41.3+/- 3.5		37.4+/- 3.6		0.03
**Leukocytes (Median +/- IQR)**		8179.2+/- 9556.7		7467.5+/- 1751.6		6950+/- 2748		7260+/- 1654.7		11670+/- 19582.3		8157.5+/- 378.2		6300+/- 1461.2		5600+/- 1838.5		5742.5+/- 705.3		7560+/- 2044.1		0.981
**Platelets (Median +/- IQR)**		251041.1+/- 75967.1		313000+/- 126649.2		249200+/- 90679.2		204500+/- 36062.5		265545.5+/- 70139		205986.3+/- 10984.7		262600+/- 70048.6		230500+/- 152028		215250+/- 55578.1		287400+/- 77190.1		0.358
**Total Cholesterol (Median +/- IQR)**		192.5+/- 38.7		206.7+/- 36.7		172.3+/- 35.4		176.2+/- 9.6		177+/- 9.3		191+/- 16.4		197+/- 54.5		122+/- 17		261.7+/- 40.1		212.1+/- 18.6		0
**HDL Cholesterol (Mean +/- SD)**		50.7+/- 11.9		56.3+/- 15.5		43.5+/- 6.2		33.4+/- 0.2		50.8+/- 5.8		53.9+/- 6.1		40.6+/- 6.2		26.5+/- 9.2		65.4+/- 10.1		62.7+/- 1.6		0
**LDL Cholesterol (Median +/- IQR)**		108.3+/- 36.5		125.6+/- 42		100.1+/- 25.6		96.7+/- 35.1		96.1+/- 9.8		107.9+/- 10.4		117.6+/- 75.4		37+/- 12.8		154+/- 23.7		117.1+/- 19.7		0.014
**VLDL Cholesterol (Median +/- IQR)**		29.1+/- 11.7		24.9+/- 6		36+/- 19		34.4+/- 2.3		30.2+/- 4.1		24.4+/- 11.6		22+/- 8.5		10.9+/- 3		42.3+/- 15.8		32.4+/- 6.8		0.021
**Triglycerides (Median +/- IQR)**		164.5+/- 91.4		124.4+/- 29.9		180+/- 94.9		171.5+/- 11.4		150.2+/- 19.8		122.4+/- 57.6		272.6+/- 201.8		54.5+/- 14.9		212+/- 79.5		174.9+/- 53.2		0.064
**Glucose (Median +/- RI)**		112.8+/- 42.9		118.8+/- 11.4		128.4+/- 58.7		184.6+/- 8.8		89.6+/- 14		90.5+/- 20.1		137.4+/- 74		155+/- 31.2		146.3+/- 32.3		82.1+/- 8.4		0.002

### Taxonomic composition of the gut microbiota

To provide a comprehensive overview of the microbial landscape across all participants, we generated a heat map with hierarchical clustering based on the relative abundance of bacterial genera. This unsupervised analysis, which included all samples without stratification by region, age, or BMI, allowed us to visualize global distribution patterns and identify taxa enriched in specific subsets of individuals. The heat map complements the bar plot and diversity analyses by highlighting the marked interindividual variability, where most genera remained at low abundance while a few taxa displayed localized peaks across the cohort.

The dendrogram analysis allowed the identification of sample clusters characterized by specific genera. In particular, one cluster was associated with *Segatella*, a genus previously identified as dominant across several subgroups of the cohort. Similarly, a second cluster showed enrichment in *Succinivibrio*, consistent with the stratified analyses by age, where this genus was absent in normotensive individuals under 50 years but present in other conditions, mainly hypertensive patients. These observations suggest that certain genera may contribute to the differentiation of patient subgroups within the cohort ( Figure 1).

**Figure 1.**
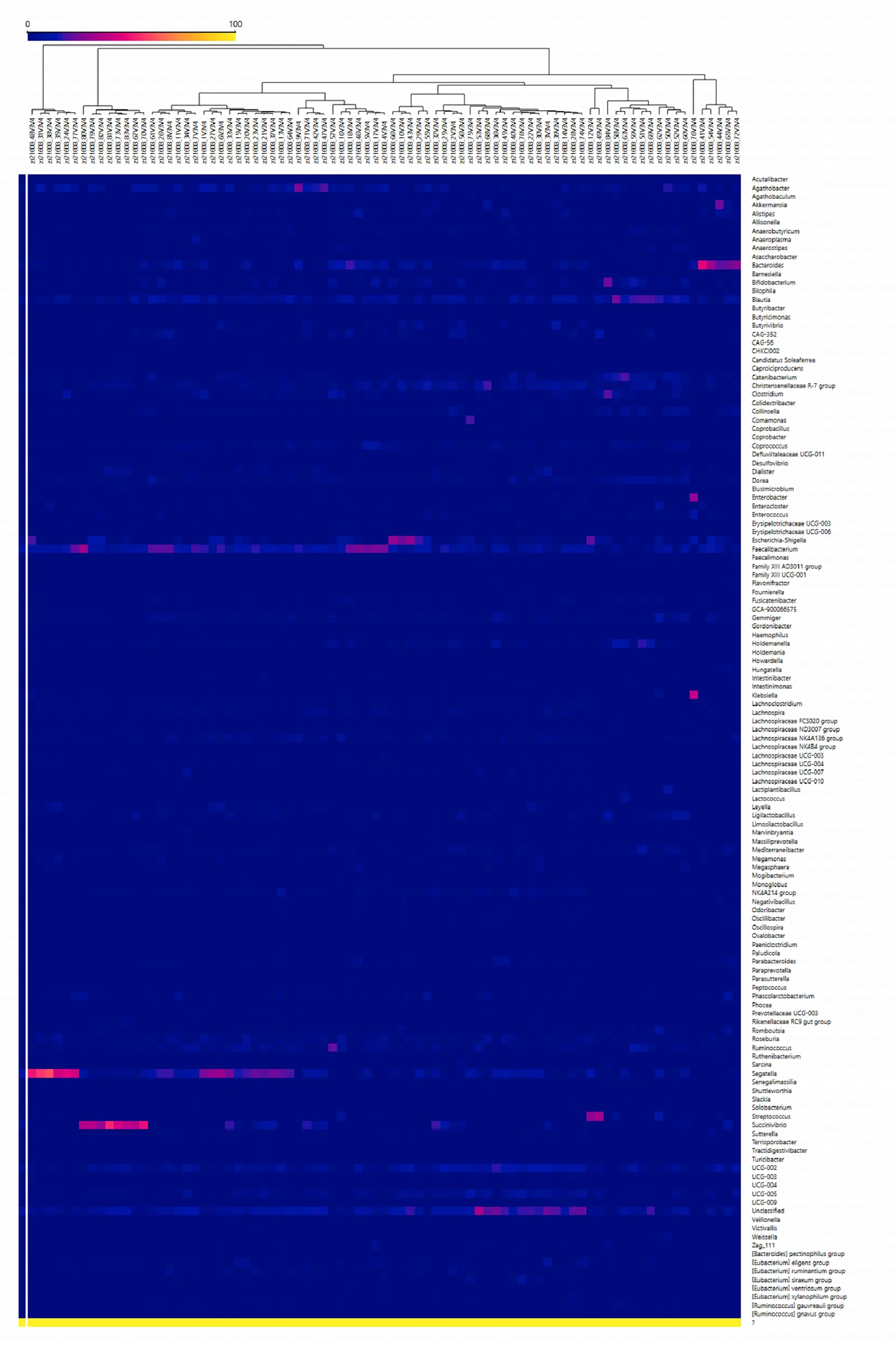
Relative abundances of bacterial genera are represented by a color gradient (blue = low abundance, yellow = high abundance). Rows correspond to genera and columns to individual samples. Hierarchical clustering of samples (top dendrogram) highlights patterns of similarity in microbial composition across the cohort.

### Microbiota composition by body mass index (BMI)

The comparative analysis of bacterial community composition at the phylum level revealed distinct differences across the four experimental conditions (H0 < 25, H0 ≥ 25, H1 < 25, H1 ≥ 25). *Bacillota* exhibited the highest mean relative abundance in all conditions, followed by *Bacteroidota. Actinomycetota* and *Pseudomonadota* were detected at intermediate abundances, whereas *Verrucomicrobiota* and the group of phyla with relative abundance < 1% remained consistently low across all comparisons. Notable shifts in the relative proportions of *Bacillota* and *Bacteroidota* were observed between H0 and H1 groups, as well as between the < 25 and ≥ 25 subcategories ( Figure 2).

**Figure 2.**
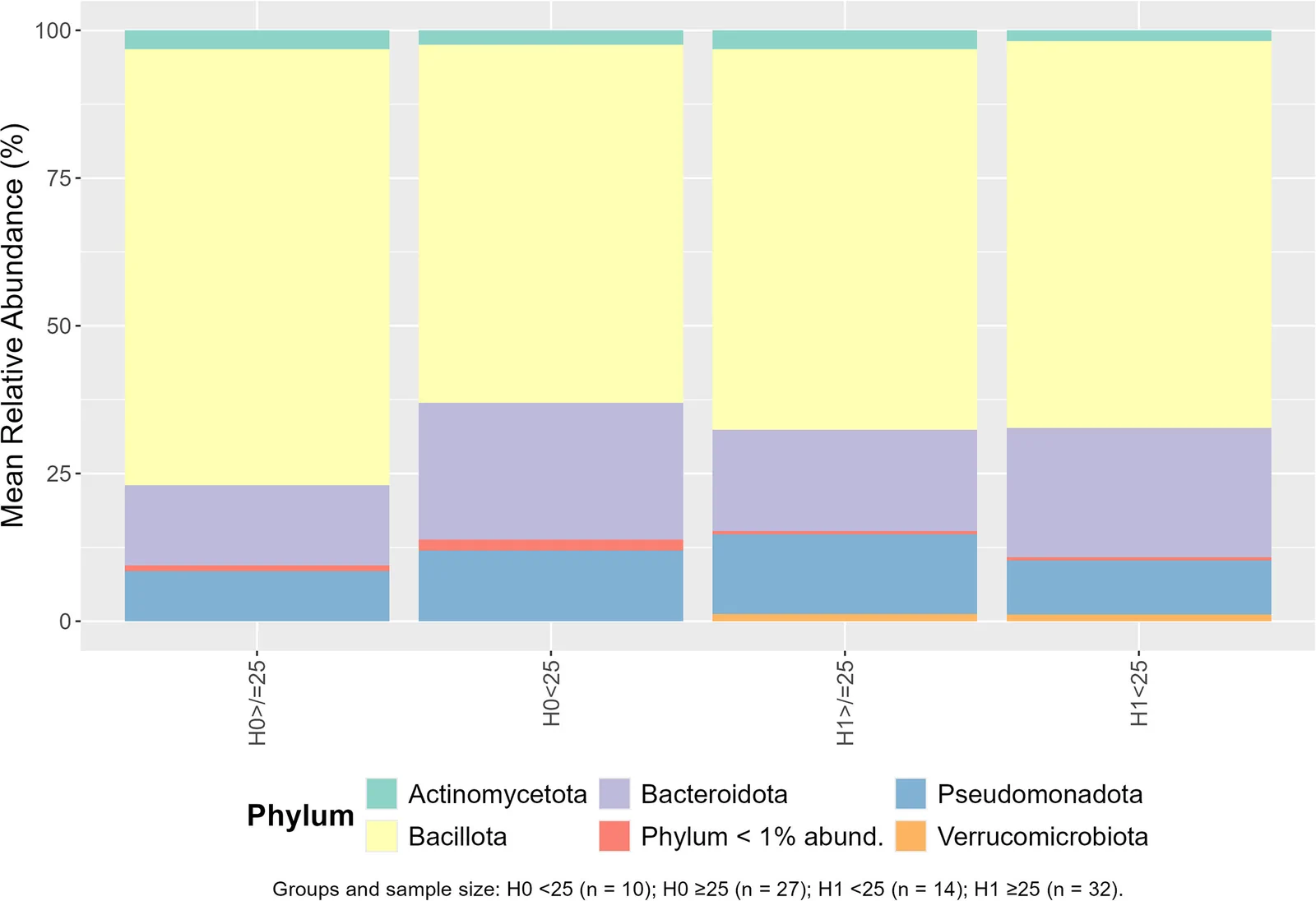
Mean relative abundance of bacterial phyla in intestinal microbiota according to BMI category (<25 or ≥25) and hypertension status (H0: normotensive; H1: hypertensive).

Genus-level profiling of the bacterial communities revealed notable differences in composition between the four experimental conditions (H0 < 25, H0 ≥ 25, H1 < 25, H1 ≥ 25). The genera *Blautia,* CAG-352, *Segatella, Succinivibrio* and UCG-002 displayed the highest mean relative abundances across conditions. The genus *Alistipes* was detected only in the H0 < 25. In the H1 group, *Ligilactobacillus* was absent. Likewise, the genera *Holdemanella*, *Clostridium*, and *Gemmiger* were not present in H0 < 25. On the other hand, *Streptococcus* was found exclusively in H1 > 25 ( Figure 3).

**Figure 3.**
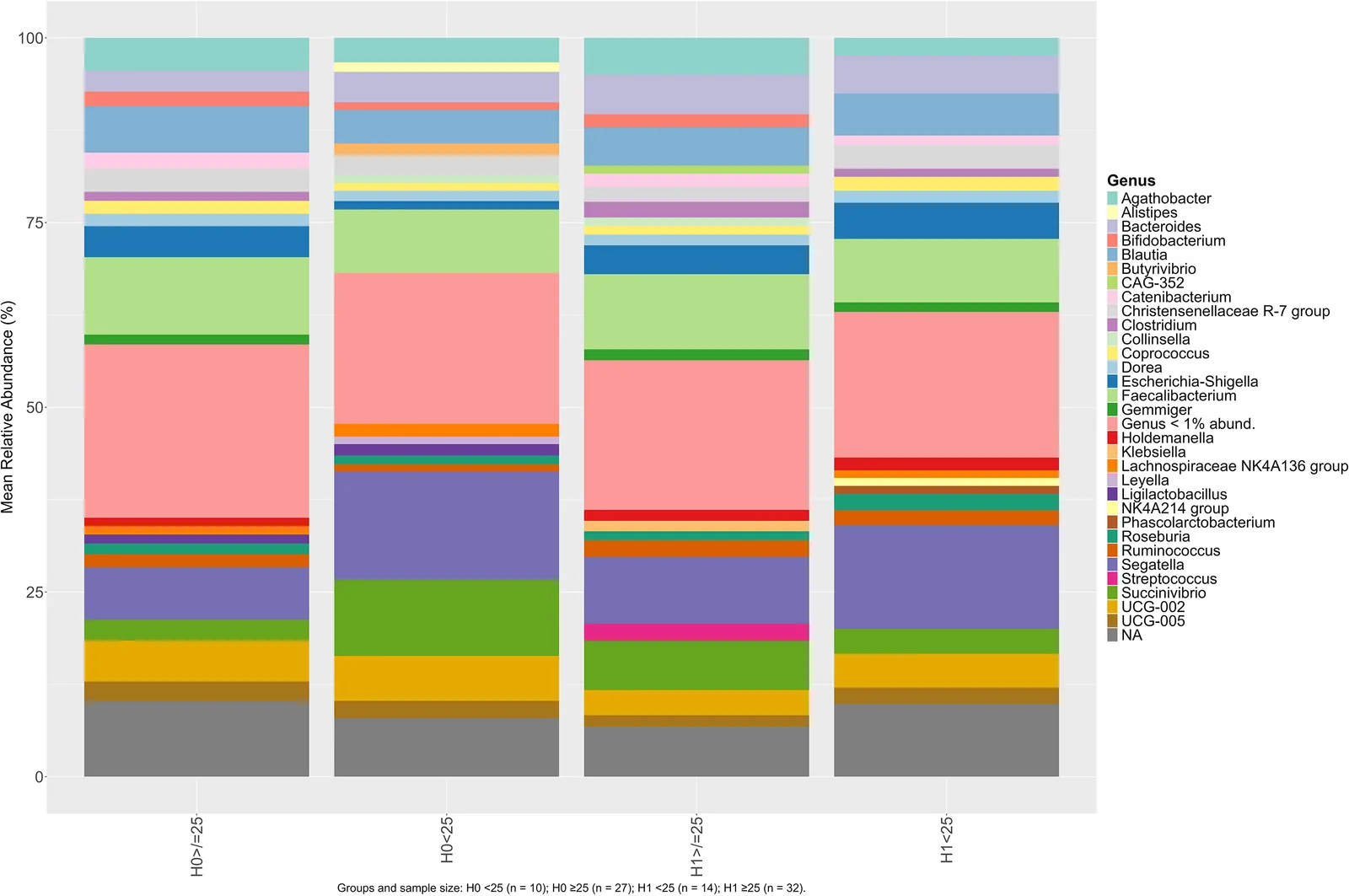
Mean relative abundance of bacterial genera in intestinal microbiota according to BMI category (<25 or ≥25) and hypertension status (H0: normotensive; H1: hypertensive).

Alpha diversity, measured using the Shannon index, showed variation across the four experimental conditions (H0 ≥ 25, H0 < 25, H1 ≥ 25, H1 < 25). Median diversity values were highest in the H1 < 25 group, followed by H0 < 25 and H1 ≥ 25, while the H0 ≥ 25 group exhibited the lowest median value. The interquartile ranges indicated moderate variability within each group, with some degree of overlap across conditions ( Figure 4).

**Figure 4.**
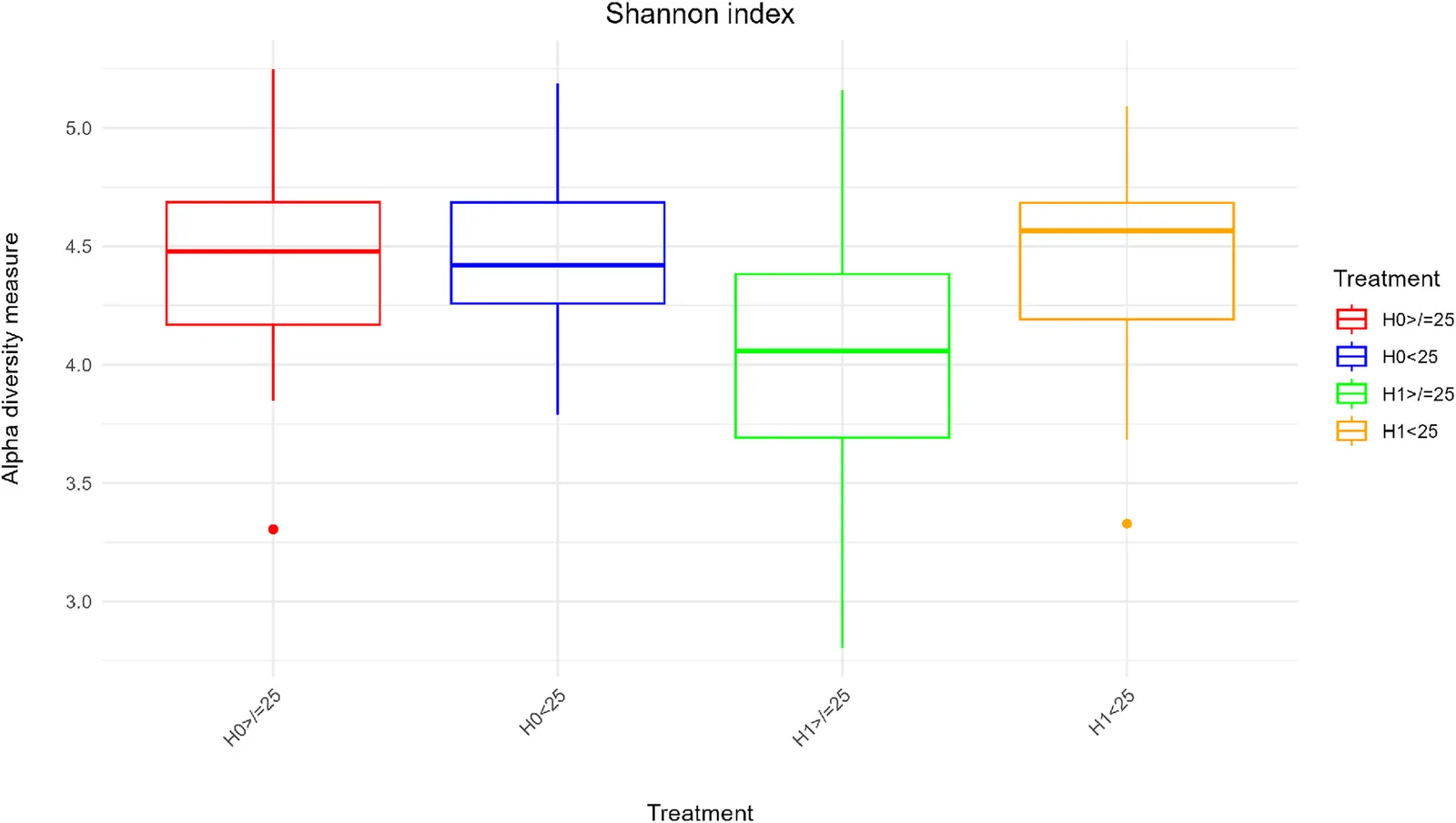
Alpha diversity of intestinal microbiota, measured using the Shannon index, according to BMI category (<25 or ≥25) and hypertension status (H0: normotensive; H1: hypertensive).

Non-metric multidimensional scaling (NMDS) based on weighted UniFrac distances (stress = 0.2334) showed substantial overlap among the four experimental conditions, with no clear separation between hypertensive and normotensive groups or between BMI categories along either NMDS axis ( Figure 5).

**Figure 5.**
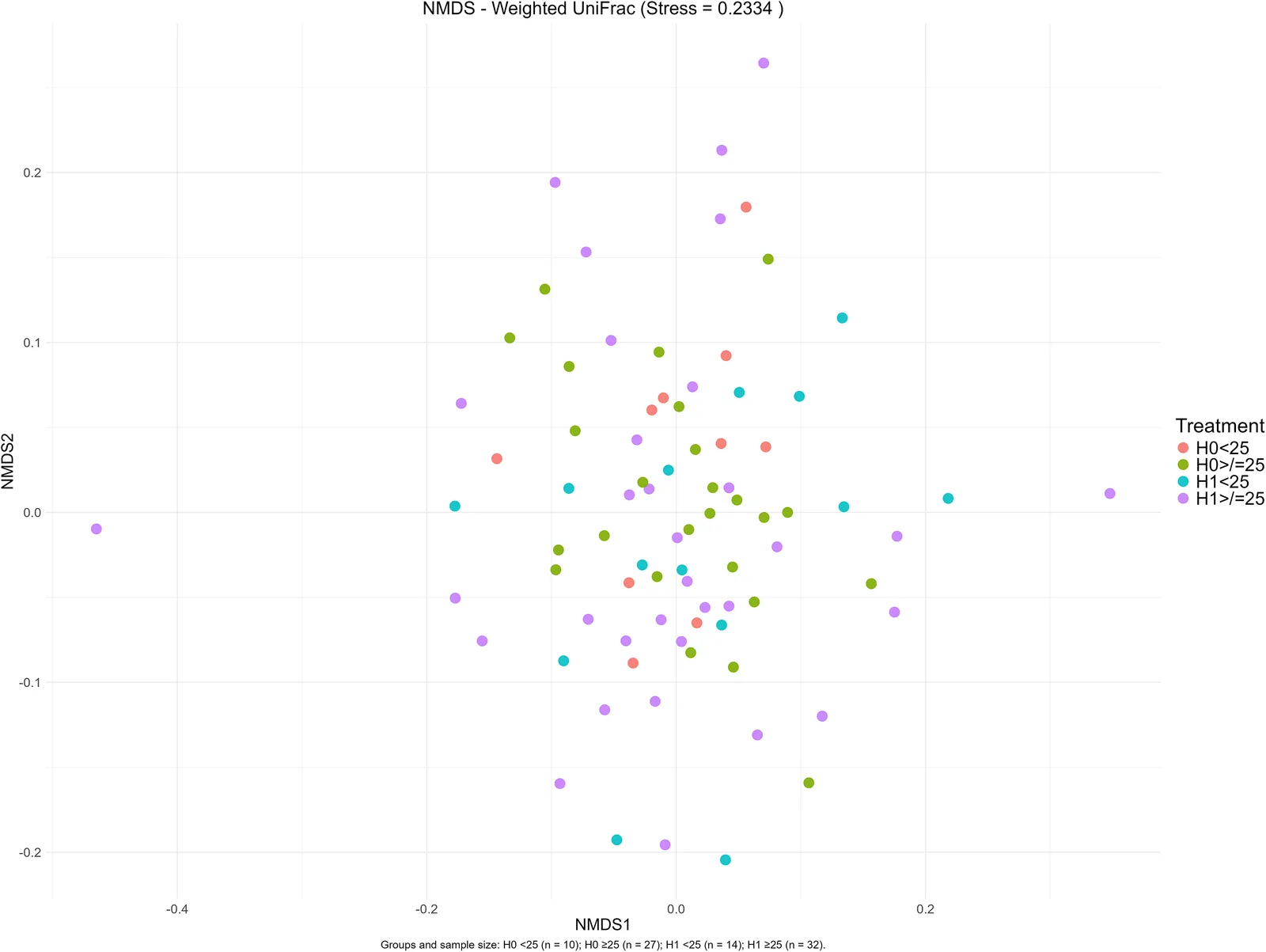
Non-metric multidimensional scaling (NMDS) plot based on weighted UniFrac distances showing beta diversity of intestinal microbiota according to BMI category (<25 or ≥25) and hypertension status (H0: normotensive; H1: hypertensive). Each point represents an individual sample, colored by group: H0 <25, H0 ≥25, H1 <25, and H1 ≥25.

### Microbiota composition by age

At the phylum level, bacterial community composition differed across the four experimental conditions defined by H grouping and age category (< 50 vs. ≥ 50 years). *Bacillota* represented the most abundant phylum in all groups, followed by *Bacteroidota.* *Actinomycetota* and *Pseudomonadota* occurred at intermediate levels, whereas *Verrucomicrobiota* and the aggregate of phyla with < 1% mean relative abundance remained consistently low ( Figure 6).

**Figure 6.**
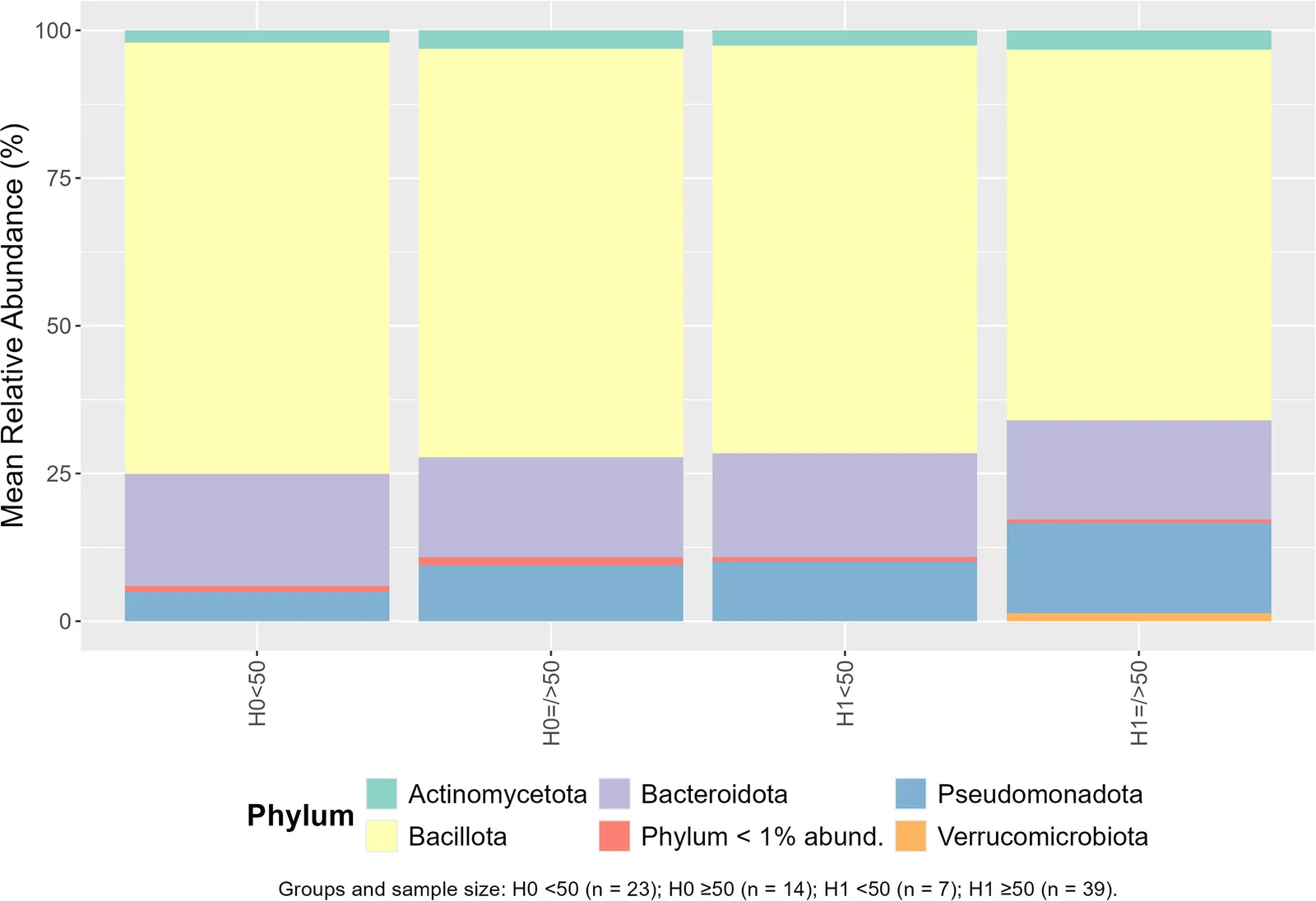
Mean relative abundance of bacterial phyla in intestinal microbiota according to age group (<50 or ≥50 years) and hypertension status (H0: normotensive; H1: hypertensive).

Genus-level analysis of bacterial communities, stratified by the H grouping and the age threshold (< 50 vs. ≥ 50 years), revealed consistent dominance *Segatella, Faecalibacterium* and *Blautia* across all conditions. It is worth noting that the genera *Collinsella* and *Streptococcus* are only present in H1, *Klebsiella* is only present in H1 over 50, and *Succinivibrio* is absent in H0 under 50 ( Figure 7).

**Figure 7.**
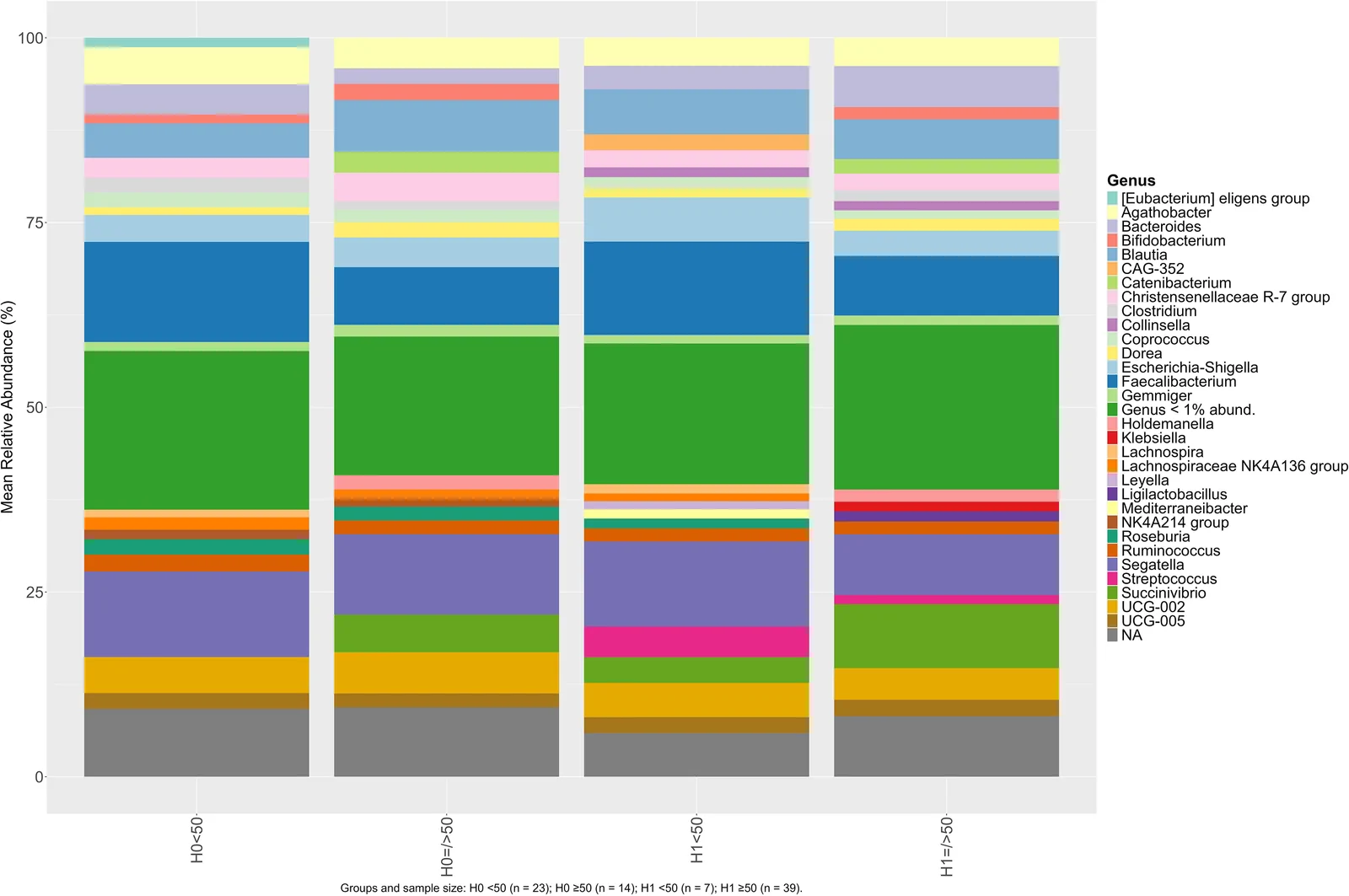
Mean relative abundance of bacterial genera in intestinal microbiota according to age group (<50 or ≥50 years) and hypertension status (H0: normotensive; H1: hypertensive).

Alpha diversity, assessed using the Shannon index, varied across the four experimental conditions defined by H grouping and age category (< 50 vs. ≥ 50 years). Median values were highest in the H1 < 50 group, followed by H0 < 50 and H1 ≥ 50, whereas the H0 ≥ 50 group exhibited the lowest median diversity. The interquartile ranges indicated moderate dispersion within each group, with some degree of overlap among conditions. These observations suggest potential differences in microbial diversity related to both H grouping and age, with higher diversity generally observed in the younger subgroups, particularly under the H1 condition ( Figure 8).

**Figure 8.**
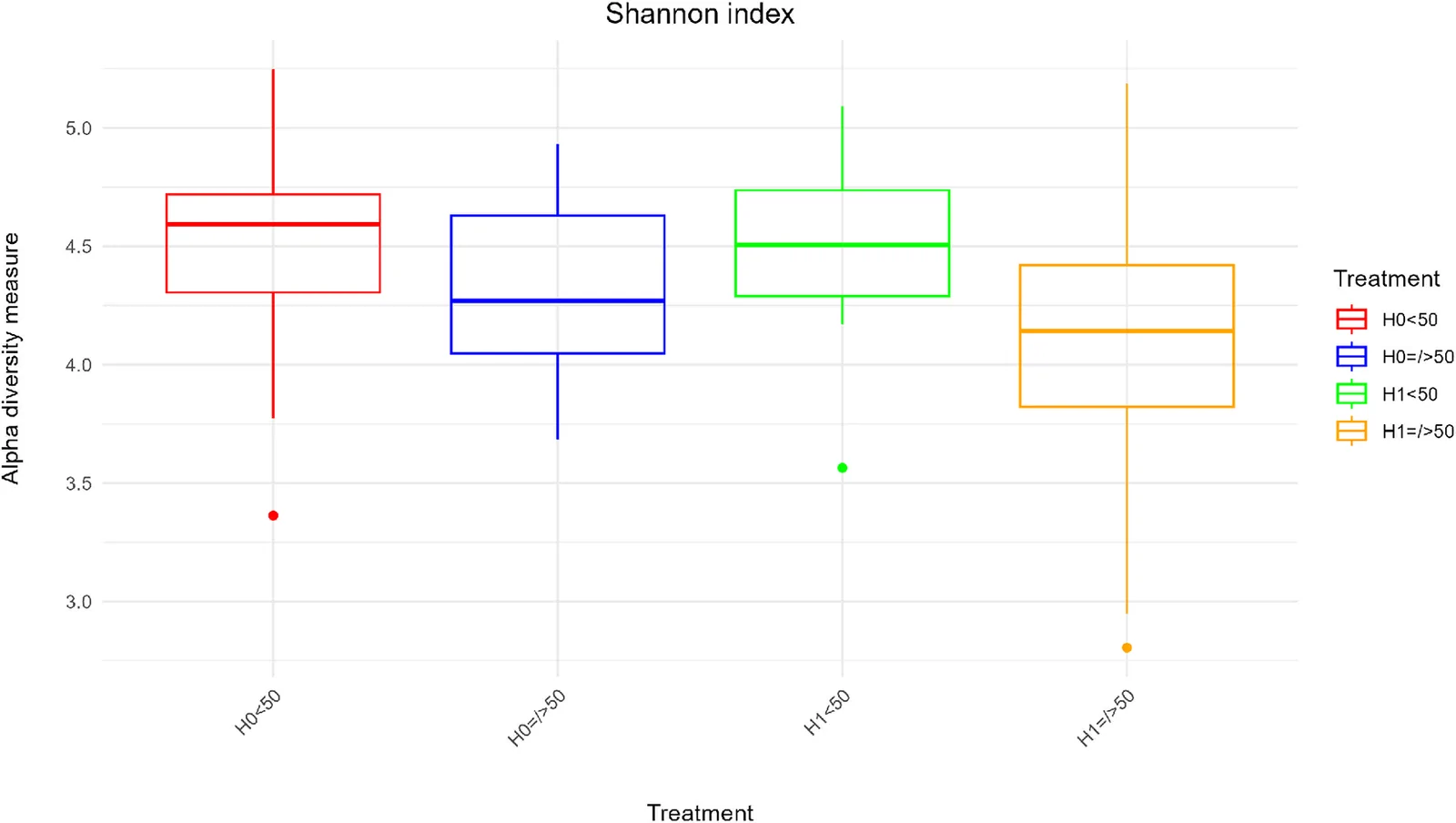
Alpha diversity of intestinal microbiota, measured using the Shannon index, according to age group (<50 or ≥50 years) and hypertension status (H0: normotensive; H1: hypertensive). Boxplots show the median, interquartile range, and outliers for each group: H0 ≥ 50, H0 < 50, H1 ≥ 50, and H1 < 50.

Non-metric multidimensional scaling (NMDS) based on weighted UniFrac distances (stress = 0.2392) showed substantial overlap among the four experimental conditions defined by H grouping and age category (<50 vs. ≥50 years), with no clear separation between hypertensive and normotensive groups or between age categories along either NMDS axis, suggesting that neither H grouping nor age was strongly associated with overall community-level differences when abundance and phylogenetic relationships are taken into account ( Figure 9).

**Figure 9.**
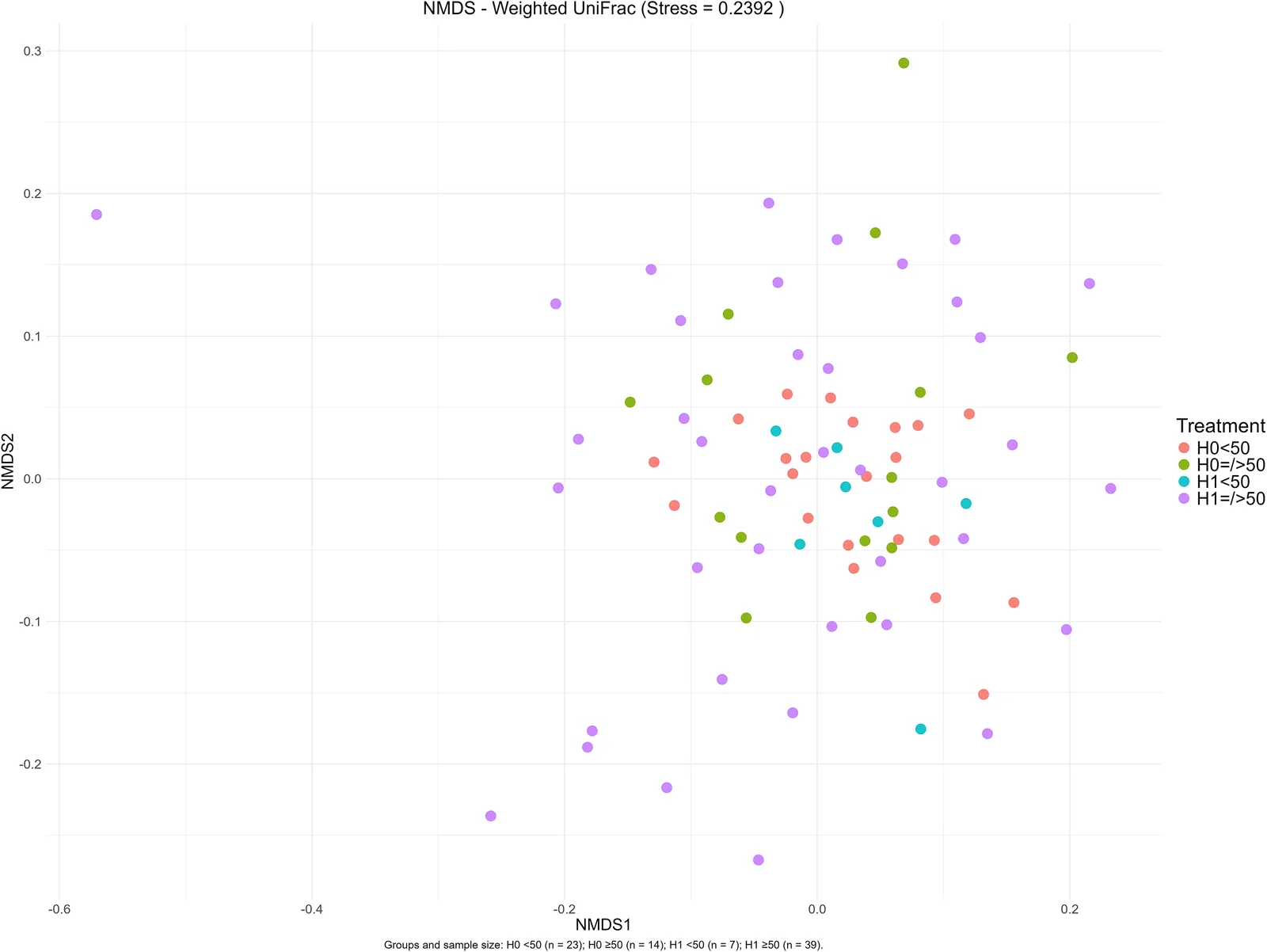
Non-metric multidimensional scaling (NMDS) plot based on weighted UniFrac distances showing beta diversity of intestinal microbiota according to age group (<50 or (≥50 years) and hypertension status (H0: normotensive; H1: hypertensive). Each point represents a sample, colored by group: H0 <50, H0 ≥50, H1 <50, and H1 ≥50.

### Microbiota composition by localization

The mean relative abundance of bacterial phyla varied among sampling locations and between conditions (H0 and H1). Across all sites *Bacillota*, *Bacteroidota*, and *Pseudomonadota* were consistently detected as dominant groups. The relative contribution of each phylum differed by location, with noticeable shifts in the proportional representation of dominant taxa between conditions ( Figure 10).

**Figure 10.**
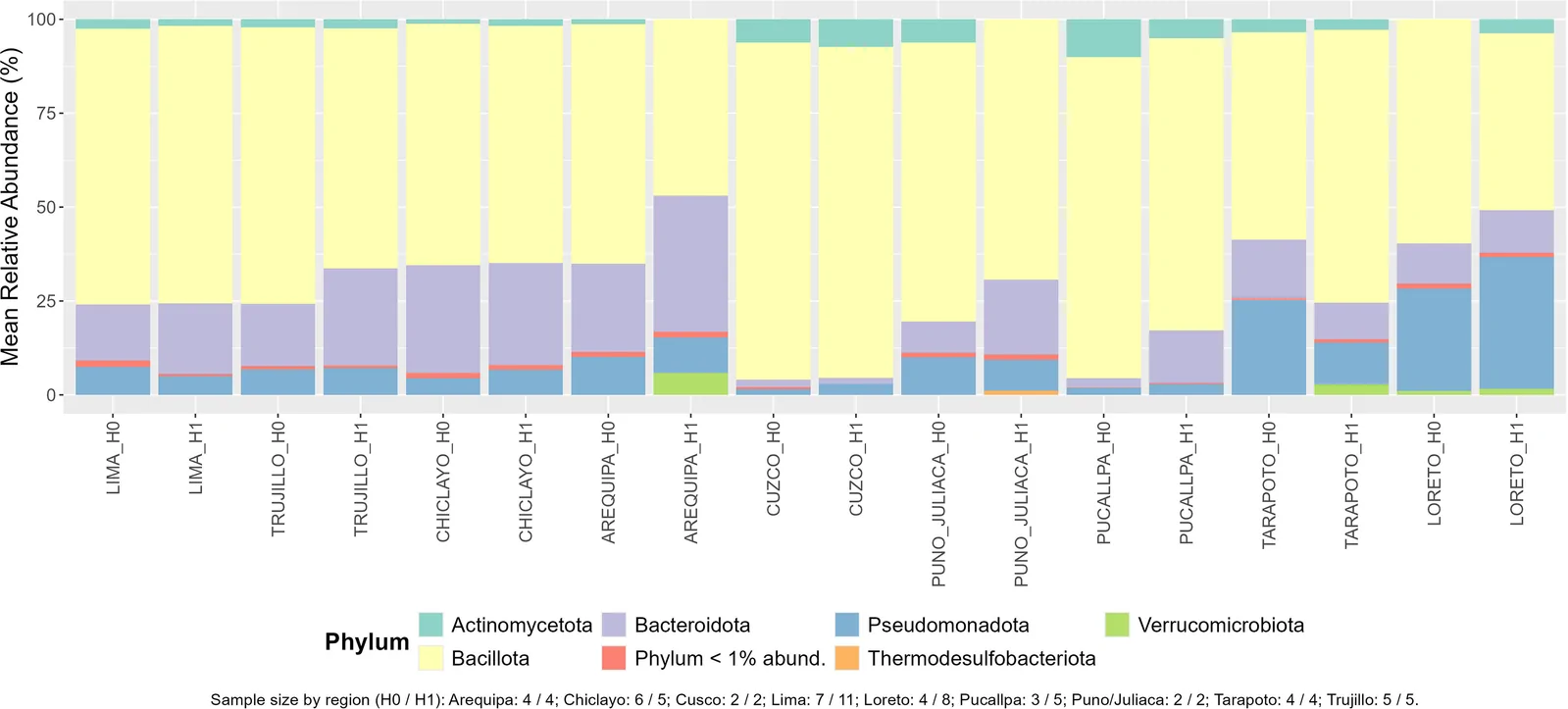
Mean relative abundance of bacterial phyla in intestinal microbiota across different Peruvian cities, stratified by hypertension status (H0: normotensive; H1: hypertensive).

The mean relative abundance of bacterial genera exhibited marked variation among sampling locations and between conditions (H0 and H1). The genera *Lachnospiraceae*, *UCG-002*, *Escherichia–Shigella*, *Enterobacter*, and *Bacteroides* were present in the majority of patients, regardless of location. A greater number of different genera was observed in the H0 groups, irrespective of location. Notably, *Streptococcus* showed relative abundances above 10% in Cuzco H1, while *Bacteroides* exceeded this threshold in Juliaca and Arequipa H1. *Succinivibrio* was recorded in both H0 and H1 groups from Tarapoto and Loreto, with abundance values reaching up to 20%. Additionally, Trujillo (H0 and H1) and Chiclayo (H0 and H1) exhibited high abundances of *Sutterella* ( Figure 11).

**Figure 11.**
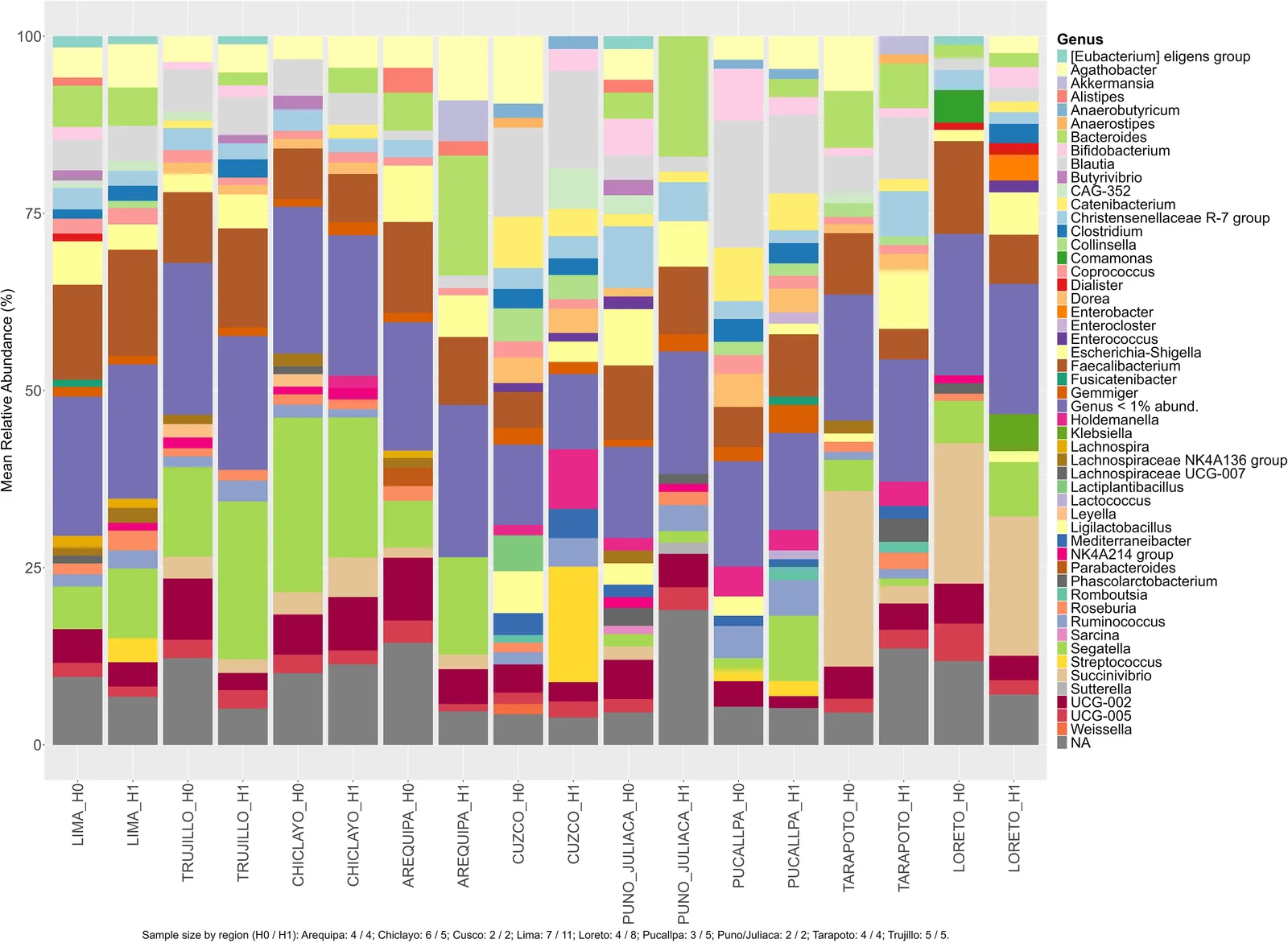
Mean relative abundance of bacterial genera in intestinal microbiota across different Peruvian cities, stratified by hypertension status (H0: normotensive; H1: hypertensive).

The Shannon diversity index values ranged approximately from 3.0 to 5.0 across sampling locations and conditions (H0 and H1). Higher diversity values were generally observed in certain Andean and coastal sites, while some Amazonian locations exhibited comparatively lower indices. Variation between conditions within the same location was evident in multiple cases, with changes in Shannon index magnitude suggesting differences in alpha diversity between treatments ( Figure 12).

**Figure 12.**
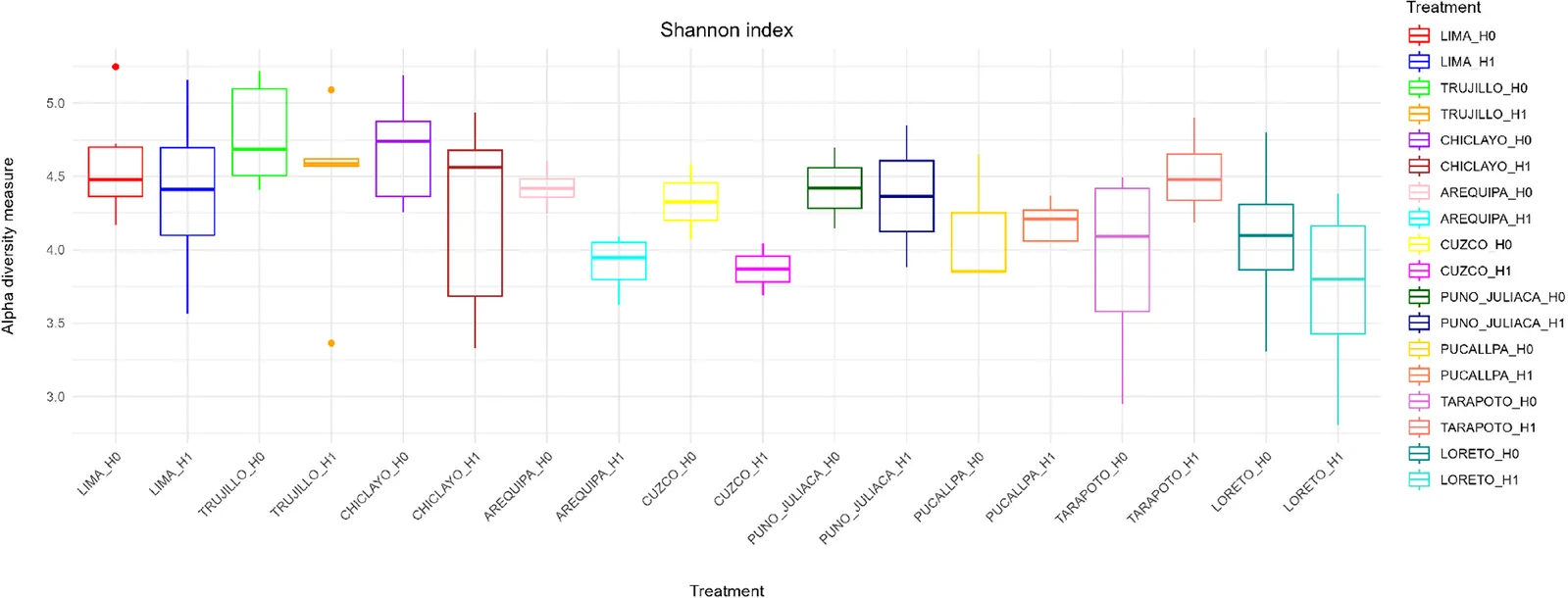
Alpha diversity of intestinal microbiota, measured using the Shannon index, across different Peruvian cities and stratified by hypertension status (H0: normotensive; H1: hypertensive). Boxplots display the median, interquartile range, and outliers, showing variation in diversity levels between locations and health status groups.

Non-metric multidimensional scaling (NMDS) based on weighted UniFrac distances (stress = 0.2547) showed substantial overlap among samples from different Peruvian cities and hypertension status groups, with no clear separation, suggesting that neither geographic location nor hypertension status was strongly associated with distinct community-level differences in this ordination ( Figure 13).

**Figure 13.**
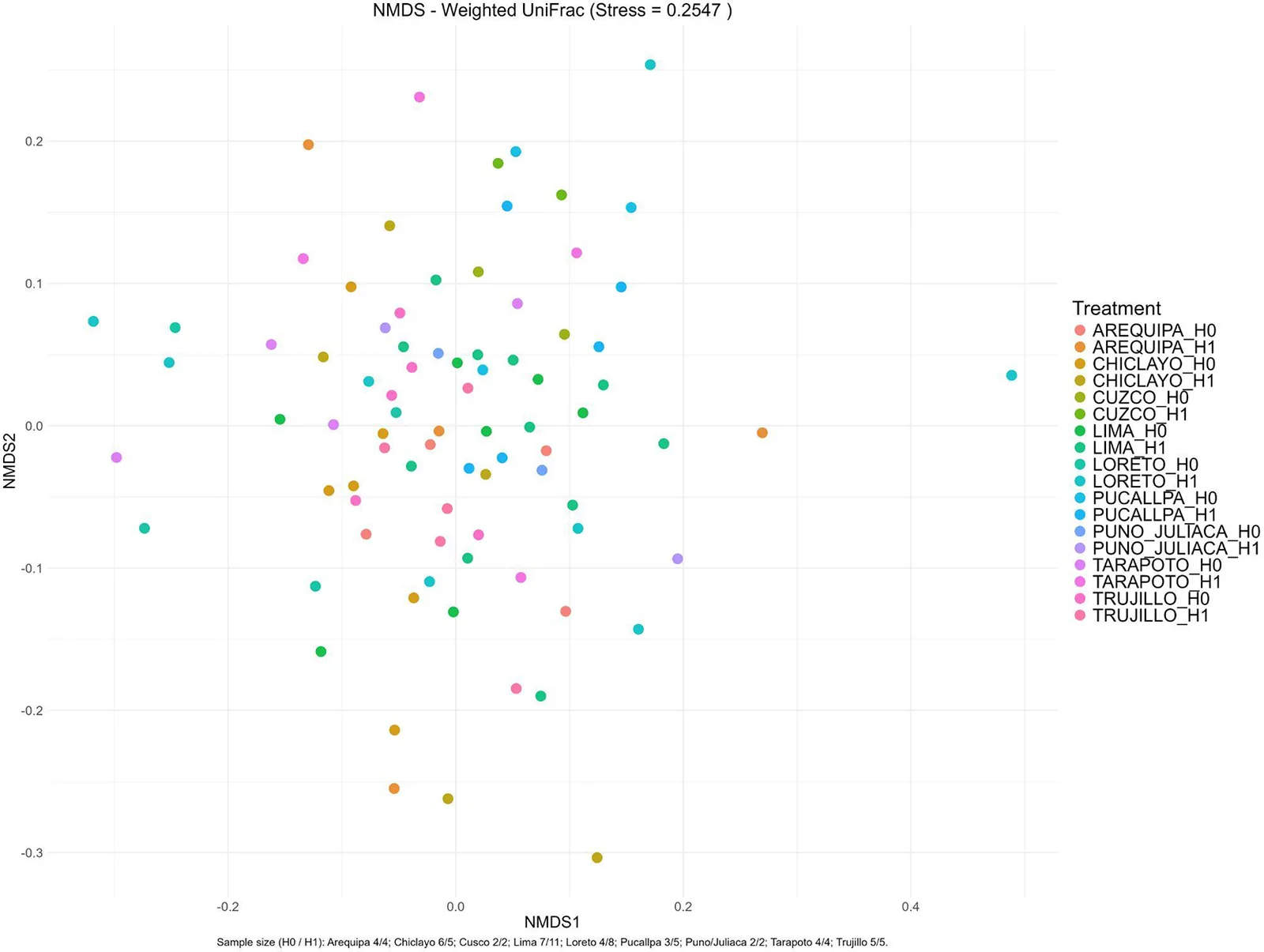
Non-metric multidimensional scaling (NMDS) plot based on weighted UniFrac distances showing beta diversity of intestinal microbiota across different Peruvian cities and hypertension status (H0: normotensive; H1: hypertensive).

### Functional predictions

Functional analysis with PICRUSt2 revealed consistent metabolic profiles across regions and conditions. The most abundant pathways included carbohydrate metabolism, amino acid metabolism, metabolism of cofactors and vitamins, energy metabolism, nucleotide metabolism, glycan biosynthesis, lipid metabolism, metabolism of other amino acids, biosynthesis of secondary metabolites, terpenoid/polyketide metabolism, and xenobiotics biodegradation. These patterns were stable across hypertensive and normotensive groups ( Figure 14).

**Figure 14.**
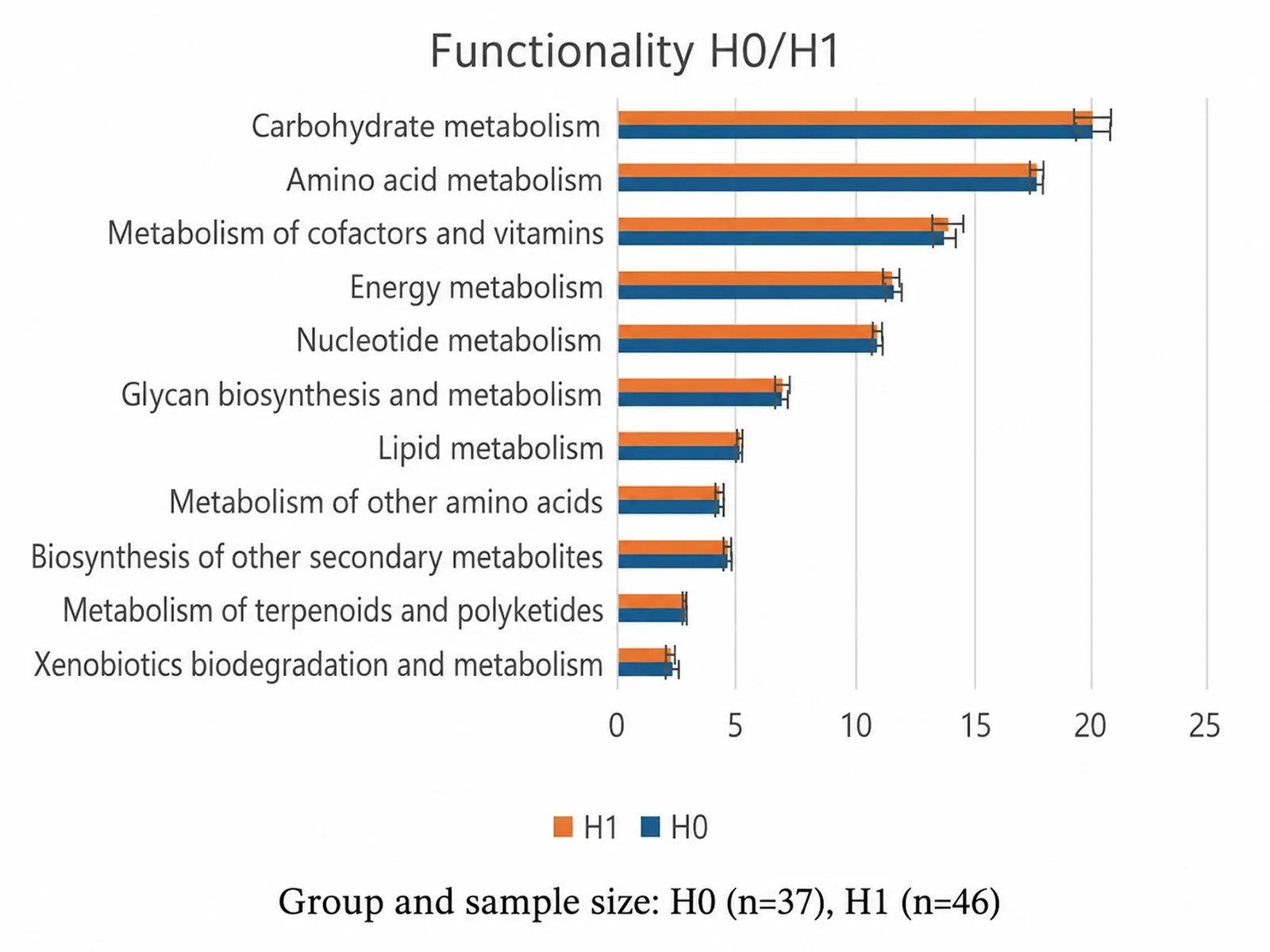
Predicted functional profiles of gut microbiota in hypertensive (H1) and normotensive (H0) individuals. The bar chart shows the relative abundance (%) of major metabolic pathways inferred from 16S rRNA gene sequencing data using functional prediction analysis.

Although no significant differences were observed between hypertensive and normotensive groups, functional shifts were detected by BMI and age. For example, individuals ≥50 years and those with BMI ≥25 showed distinct contributions of amino acid and lipid metabolism compared to their counterparts. These findings suggest that host-related factors may drive functional variation more strongly than hypertension status itself.

### Adjusted multivariable analysis

To determine whether hypertension status remained associated with microbiota composition after accounting for region, age, and BMI, we performed adjusted PERMANOVA and multiple linear regression models using the 83 samples with available sequencing data (see Methods). In the adjusted PERMANOVA on Bray-Curtis dissimilarities, region explained the largest share of variance in overall community composition (R ^2^ = 16.3%, pseudo-F = 1.81, p = 0.001) and age group was marginally associated (R ^2^ = 1.6%, pseudo-F = 1.43, p = 0.043), whereas BMI group (R ^2^ = 0.8%, p = 0.925) and hypertension status (R ^2^ = 1.1%, pseudo-F = 0.97, p = 0.510) were not significantly associated with beta diversity once the other factors were accounted for. Similarly, in the adjusted linear model for Shannon alpha diversity, hypertension status was not significantly associated with diversity (p = 0.259), nor were age (p = 0.384) or BMI (p = 0.442); region approached but did not reach statistical significance (p = 0.053). These adjusted analyses indicate that the associations between hypertension and microbiota features described above are not statistically robust once region, age, and BMI are accounted for, and that regional variation is the strongest independent correlate of overall community composition in this cohort.

## Discussion

This study investigated the composition, diversity, and functional potential of the gut microbiota in hypertensive and normotensive adults from nine regions of Peru, integrating demographic, anthropometric, and geographical factors. To our knowledge, this is one of the first studies in Latin America, and the first in Peru, to examine the gut microbiota in relation to arterial hypertension using high-throughput sequencing. Our findings provide novel insights into the interplay between host factors and microbial communities in a highly diverse population characterized by unique dietary, cultural, and environmental conditions. The regional breakdown of hypertensive patients further revealed heterogeneity in age, BMI, and parasitic infection rates across Peru. Coastal regions contributed the largest share of hypertensive cases, while jungle areas showed a higher prevalence of intestinal parasites, conditions that may additionally shape gut microbiota composition. These variations highlight that hypertension in Peru coexists with region-specific nutritional and environmental exposures, reinforcing the need to interpret microbiota findings within their local context rather than in isolation from broader determinants of health.

### Microbiota composition in hypertensive versus normotensive individuals

Across all participants, the gut microbiota was dominated by the phyla *Bacillota* and *Bacteroidota*, consistent with global reports that identify these two groups as central to human gut ecology. ^16, 17^ At the genus level, *Blautia*, *CAG-352*, *Segatella*, and *Succinivibrio* were abundant across groups, while specific taxa distinguished hypertensives from normotensives. In particular, *Streptococcus* was enriched in hypertensives with BMI ≥25, whereas *Alistipes* was detected only in normotensives with BMI <25. These results align with previous studies reporting *Streptococcus* and cardiovascular risk, ^18, 19^ and strains of *Alistipes* are associated with lower blood pressure variability. ^20, 21^ Hypertensive patients showed a lower relative abundance of the genus *Ligilactobacillus.* This observation is in line with experimental data in spontaneously hypertensive rats, where *Ligilactobacillus murinus* was markedly reduced and its supplementation led to significant improvements in vascular and intestinal health. Specifically, oral administration of *L. murinus* lowered blood pressure, improved endothelial function, restored tight junction integrity, and reduced plasma endotoxin levels in hypertensive animals. Taken together, these findings suggest that the reduced abundance of *Ligilactobacillus* in our Peruvian cohort may not only serve as a microbial signature associated with hypertension but also highlight this genus as a potential target for probiotic-based interventions aimed at improving cardiovascular and intestinal health. ^22^ Beyond these genera, the heatmap clustering analysis provided additional insights into taxa that distinguished subsets of participants.

The clustering observed in the heatmap highlighted the consistent presence of *Segatella* and a separate cluster enriched in *Succinivibrio.* *Segatella* has been recently described as part of the *Segatella copri* complex, where species-level differences are associated with host lifestyle and diet, supporting the idea that its dominance in our cohort may reflect local dietary patterns rich in carbohydrates and fiber. ^23^ By contrast, *Succinivibrio* appeared differentially distributed, being absent in younger normotensive individuals but present in hypertensive patients. Although direct associations between *Succinivibrio* and hypertension are limited, members of this genus are recognized succinate producers in the gut. Elevated circulating succinate has been reported in obesity, hypertension, and metabolic disease, and has been mechanistically linked to pro-inflammatory signaling and endothelial dysfunction. ^24, 25^ Furthermore, succinate signaling through its receptor (SUCNR1) has been proposed as a pathway connecting microbial metabolism with blood pressure regulation and cardiovascular risk. ^26^

### Diversity patterns

In this study, we did not observe statistically significant differences in alpha diversity between hypertensive and normotensive participants. Although some trends were apparent, such as higher diversity in hypertensives with BMI <25 and those younger than 50 years, and lower values in normotensives with higher BMI or older age, these differences did not reach statistical significance. This suggests that, in our cohort, hypertension status alone may not be a strong determinant of microbial richness and evenness.

These findings contrast with several reports in both humans ^27^ and animal models that describe reduced microbial diversity as a hallmark of hypertension and other cardiometabolic disorders. ^28^ One possible explanation is that BMI and age exert stronger influences on microbial diversity than blood pressure status, as suggested by the trends observed in our subgroup analyses. Another possibility is that the relatively modest sample size limited our power to detect subtle differences.

Beta diversity analyses similarly showed only partial clustering by hypertension status and age, with substantial overlap across groups and no statistically significant separations. This reinforces the notion that community structure is broadly shared between hypertensive and normotensive individuals in this population. Taken together, these results highlight that, while reduced microbial diversity has been proposed as a microbial signature of hypertension, in our cohort the effect was not significant and appears to be modulated by host-related and environmental factors such as BMI, age, diet, and geography. ^29– 31^

### Influence of BMI and age

Our stratified analyses revealed BMI- and age-related shifts in microbial composition. For instance, *Collinsella* and *Streptococcus* were restricted to hypertensives, while *Klebsiella* was identified exclusively in hypertensives over 50 years. Previous studies have linked *Collinsella* to pro-inflammatory metabolic activity, including enhanced gut permeability and endotoxin production, which may exacerbate hypertension. ^32^ The enrichment of *Klebsiella* in older hypertensives observed in our study is concerning, as this genus has been reported to be overrepresented in pre-hypertensive and hypertensive individuals. Beyond serving as a microbial marker, experimental evidence suggests that *Klebsiella* may actively contribute to hypertension through mechanisms such as lipopolysaccharide-induced inflammation and the production of metabolites like TMAO, which impair vascular function. These findings raise the possibility that the presence of *Klebsiella* is not merely associative but may play a causal role in hypertension pathophysiology. ^25^

In contrast, *Succinivibrio* was absent in normotensive individuals under 50 years, suggesting that younger, healthier participants may maintain lower levels of this genus. This observation is particularly relevant given that succinate, a metabolite produced by several bacterial taxa including *Succinivibrio*, has been linked to obesity, insulin resistance, and hypertension, with elevated circulating levels correlating with impaired metabolic profiles and increased cardiovascular risk. ^24^ Taken together, these findings reinforce the notion that the expansion of succinate-producing bacteria such as *Succinivibrio* may contribute to host metabolic stress and highlight the complex interplay between host physiology, diet, and microbiota-derived metabolites in the pathophysiology of hypertension. These findings reinforce the complex interaction between host physiology, lifestyle, and microbiota composition.

### Geographical differences

One of the strengths of this study lies in its multi-regional design. We observed clear site-specific patterns in microbial composition. These findings suggest that geography, diet, and cultural practices shape the microbiota in ways that transcend hypertension status. ^33^ High-altitude regions, for example, are characterized by traditional diets rich in tubers and grains, while Amazonian diets emphasize cassava, plantain, and freshwater fish. Such dietary variation may explain the regional enrichment of genera with specific metabolic capacities. These findings are consistent with prior work in Peru, where subsistence strategies among the Matses (hunter-gatherers in the Amazon) and Tunapuco (traditional Andean farmers) were shown to produce microbiome profiles distinct from urban populations, enriched in taxa such as *Prevotella* and *Treponema*, linked to carbohydrate and fiber metabolism. ^34^ Moreover, ancestral Andean farming technologies, such as terraces, waru-waru, and qochas, sustain highly diverse and micronutrient-rich agroecosystems, supporting diets with a wide variety of tubers, grains, and legumes. ^35^ Importantly, these results highlight that microbiome–hypertension interactions cannot be generalized globally, and that local dietary traditions and ecological knowledge are essential for interpretation.

### Functional predictions

Functional analysis using PICRUSt2 revealed consistent metabolic pathways across groups, with predominant functions in carbohydrate, amino acid, lipid, and energy metabolism. Interestingly, no significant differences were observed between hypertensive and normotensive participants, suggesting that functional redundancy within the microbiome may buffer compositional shifts. This resilience is well described in microbial ecology, where taxonomic variation does not always translate into altered functional capacity. ^36, 37^

Nonetheless, BMI and age influenced functional predictions. Participants ≥50 years and those with BMI ≥25 showed distinct patterns in amino acid and lipid metabolism, pathways previously implicated in blood pressure regulation. For example, altered amino acid metabolism may affect nitric oxide bioavailability, while lipid metabolism disturbances could contribute to systemic inflammation and endothelial dysfunction. ^38^ These findings support the hypothesis that host factors may shape microbial function more strongly than hypertension status itself.

### Public health implications

If confirmed in larger, adequately powered studies, our findings could have implications for public health. At the regional level, they highlight the need to consider high-altitude and Amazonian populations separately when designing nutritional and microbial interventions. At the national level, the results support the development of health policies tailored to Peru’s cultural and geographic diversity. At the global level, our study contributes to growing evidence that the gut microbiome is intricately linked to cardiovascular health, but that interventions must be context-specific. Strategies such as dietary counseling, probiotic supplementation, or microbiome-targeted therapies are unlikely to succeed if they ignore local dietary traditions and environmental determinants.

## Limitations and future directions

Several limitations should be acknowledged. First, the cross-sectional design prevents causal inference, and longitudinal studies are necessary to establish whether observed microbial changes are a cause or consequence of hypertension. Second, the modest sample size may limit statistical power. Third, functional predictions based on 16S rRNA data are inherently limited, and more robust metagenomic and metabolomic approaches are needed. Fourth, several factors known to influence gut microbiota independently of hypertension — antihypertensive medication class (97.8% of hypertensive participants were treated; see Results), disease duration, detailed dietary intake, and physical activity — were not fully controlled or included as covariates in the microbiota analyses, and the regional and altitude-based subgroups were too small to support well-powered comparisons; as a result, the present analyses are largely descriptive and stratified rather than based on multivariable models adjusting simultaneously for all these factors at once, and associations reported here should be interpreted as unadjusted and potentially confounded rather than as evidence of a specific microbial signature of hypertension. In addition, because a phylogenetic tree was not available for this cohort, the adjusted beta-diversity analysis reported above used Bray-Curtis dissimilarity rather than the UniFrac distances used elsewhere in this study, which does not account for phylogenetic relatedness between taxa. Fifth, no participants older than 70 years enrolled, since recruitment relied on convenience sampling at community health campaigns rather than an age quota. To partially address the resulting age-related variation, we stratified taxonomic composition, alpha diversity, and beta diversity analyses by age group (<50 vs. ≥50 years) throughout the Results, which allowed us to assess whether hypertension-related microbiota patterns held consistently across the age range actually sampled; a similar upper age bound is also common in comparable studies. Nonetheless, given known age-related changes in the gut microbiota through mechanisms such as immunosenescence and polypharmacy, findings from this cohort should not be extrapolated to adults older than 70.

Despite these limitations, this study constitutes one of the first comprehensive analyses of the gut microbiota in hypertensive patients in Latin America, and uniquely integrates geographic, anthropometric, and demographic variables.

## Conclusions

In conclusion, this exploratory, cross-sectional study suggests that the composition and diversity of the gut microbiota in Peruvian adults vary with hypertension status, BMI, age, and geographic region. Differences such as the reduced relative abundance of *Ligilactobacillus*, enrichment of *Klebsiella*, and presence of succinate-producing taxa such as *Succinivibrio* in hypertensive participants are consistent with dysbiosis patterns reported in other populations, but should be regarded as exploratory, hypothesis-generating associations rather than evidence of a causal or hypertension-specific microbial signature, given the cross-sectional design and the modest and unevenly distributed sample. Notably, adjusted PERMANOVA and linear regression models controlling for age, BMI, and region found no statistically significant independent association between hypertension status and either beta or alpha diversity, reinforcing that the taxonomic differences described above should be interpreted with caution. At the same time, regional dietary traditions and ancestral agricultural practices strongly influence microbiota composition and must be considered when interpreting any association with blood pressure. Functional predictions suggest that microbial metabolic potential is more closely related to host characteristics, such as age and BMI, than to hypertension status alone. Together, these results underscore the importance of context-specific, adequately powered approaches in microbiome research. Larger, longitudinal studies with multivariable adjustment for confounders, ideally combining shotgun metagenomics and metabolomics, are needed to confirm these associations and to establish whether they have translational relevance for hypertension prevention and management in Peru and other Latin American populations.

## Ethical considerations

This study was approved by the Ethics Committee of the Universidad Peruana Unión (UPeU), under resolution N° 2025-CEUPeU-004, Written informed consent was obtained from each participant prior to enrollment.

## Data Availability

The raw sequencing data generated and analyzed during this study have been deposited in the National Center for Biotechnology Information (NCBI) Sequence Read Archive (SRA) under the BioProject accession number **PRJNA1310654**. Zenodo. Repository HTA PMC F. https://doi.org/10.6084/m9.figshare.30775160 ^39^ This project contains the following underlying data:
• Clinical record – model. pdf • informed consent - model HTA. pdf Data is available under the terms of the CC BY 4.0.
