# Supplementary material for: Diversity and Functionality of the Gut Microbiota in Hypertensive Patients from Different Regions of Peru
Source: F1000Res. 2026 Nov 6;14:1401. Originally published 2025 Dec 15. [Version 3] doi: 10.12688/f1000research.170893.3 (PMC13752449; doi:10.12688/f1000research.170893.3)
Supplement: Peer review history

# Reviewer report: Reviewer response for version 3

**DOI:** 10.5256/f1000research.213188.r538928

**Published:** 2026-11-10

**Authors:** Juan José Contreras Mancilla

**Affiliations:** 1 Instituto Nacional de Enfermedades Neoplásicas, Lima, Peru

All my questions have been resolved.

Is the work clearly and accurately presented and does it cite the current literature?

Yes

If applicable, is the statistical analysis and its interpretation appropriate?

Partly

Are all the source data underlying the results available to ensure full reproducibility?

Partly

Is the study design appropriate and is the work technically sound?

Yes

Are the conclusions drawn adequately supported by the results?

Partly

Are sufficient details of methods and analysis provided to allow replication by others?

Partly

Reviewer Expertise:

Molecular oncology, bioinformatics, infectious diseases

I confirm that I have read this submission and believe that I have an appropriate level of expertise to confirm that it is of an acceptable scientific standard.

# Reviewer report: Reviewer response for version 2

**DOI:** 10.5256/f1000research.209655.r524952

**Published:** 2026-10-12

**Authors:** Juan José Contreras Mancilla

**Affiliations:** 1 Instituto Nacional de Enfermedades Neoplásicas, Lima, Peru

**The revised version has substantially improved compared with version 1. **The authors addressed several major concerns by clarifying the 16S-based nature of the analysis, adding adjusted multivariable analyses, reporting antihypertensive medication use, tempering the conclusions, and emphasizing the exploratory nature of the findings. The new adjusted analyses are particularly important because they show that hypertension status is not independently associated with alpha or beta diversity after accounting for age, BMI, and region, while region remains the strongest correlate of microbial community composition.

**Some interpretation remains too strong: **The Discussion still includes statements suggesting microbial signatures, probiotic targets, or possible causal roles of specific genera such as Ligilactobacillus and Klebsiella. These statements should be softened because the study is cross-sectional, based on 16S amplicon data, and not designed to establish causality or intervention targets.

**Minor terminology and formatting issues remain: **The Methods still include “16S ARNr gene sequencing,” which should be corrected to “16S rRNA gene sequencing.” Table 2 still contains “Cost” instead of “Coast.” Some figures, particularly the heatmap, remain difficult to read due to small labels and high density of information.

Is the work clearly and accurately presented and does it cite the current literature?

Yes

If applicable, is the statistical analysis and its interpretation appropriate?

Partly

Are all the source data underlying the results available to ensure full reproducibility?

Partly

Is the study design appropriate and is the work technically sound?

Yes

Are the conclusions drawn adequately supported by the results?

Partly

Are sufficient details of methods and analysis provided to allow replication by others?

Partly

Reviewer Expertise:

Molecular oncology, bioinformatics, infectious diseases

I confirm that I have read this submission and believe that I have an appropriate level of expertise to confirm that it is of an acceptable scientific standard, however I have significant reservations, as outlined above.

# Reviewer report: Reviewer response for version 2

**DOI:** 10.5256/f1000research.209655.r524951

**Published:** 2026-10-07

**Authors:** Rosa Del campo Moreno

**Affiliations:** 1 Universidad de la Rioja, Logroño, La Rioja, Spain

All my questions have been resolved

Is the work clearly and accurately presented and does it cite the current literature?

Partly

If applicable, is the statistical analysis and its interpretation appropriate?

I cannot comment. A qualified statistician is required.

Are all the source data underlying the results available to ensure full reproducibility?

Yes

Is the study design appropriate and is the work technically sound?

No

Are the conclusions drawn adequately supported by the results?

No

Are sufficient details of methods and analysis provided to allow replication by others?

Partly

Reviewer Expertise:

Microbiota and health

I confirm that I have read this submission and believe that I have an appropriate level of expertise to confirm that it is of an acceptable scientific standard.

# Reviewer report: Reviewer response for version 1

**DOI:** 10.5256/f1000research.188405.r491252

**Published:** 2026-06-22

**Authors:** Juan José Contreras Mancilla

**Affiliations:** 1 Instituto Nacional de Enfermedades Neoplásicas, Lima, Peru

**Overall recommendation**

 The study addresses an important and underexplored topic: the gut microbiota of hypertensive and normotensive adults from different regions of Peru. The regional scope and inclusion of participants from coastal, highland, and Amazonian areas are valuable. However, in its current form, the manuscript requires substantial methodological, statistical, interpretative, and reporting improvements before it can be considered scientifically sound.

**Major strengths**

**1. Relevant and understudied population.** The study focuses on Peruvian adults from multiple geographic regions, which is valuable given the underrepresentation of Latin American populations in microbiome research.

**2. Multiregional design.** Including coastal, highland, and jungle regions allows the authors to explore geographic and environmental variation, which is highly relevant for microbiome studies.

**3. Use of standard microbiome tools.** The authors used commonly applied tools such as DADA2, phyloseq, vegan, DESeq2, and PICRUSt2, which are appropriate if fully described and correctly applied.

**4. Recognition of limitations.** The manuscript appropriately acknowledges the cross-sectional design, modest sample size, and the limitations of functional prediction from 16S rRNA data.

**Major concerns** that must be addressed:

 1. The manuscript should not describe the approach as “metagenomic sequencing”

 The study used 16S rRNA gene amplicon sequencing of the V3–V4 region, not shotgun metagenomic sequencing. This distinction is important because 16S amplicon sequencing provides taxonomic profiles with limited resolution and only inferred functional potential through prediction tools such as PICRUSt2.

 This point is essential because the current wording may overstate the methodological scope of the study.

 2. Hypertension status is strongly confounded by age and potentially by other variables

 The hypertensive group was substantially older than the normotensive group. Age is a major determinant of gut microbiota composition and diversity, and therefore unadjusted comparisons between hypertensive and normotensive participants are difficult to interpret. BMI, region, parasitic infection, medication use, diet, and sample storage conditions may also confound the observed microbiome patterns.

 The current analysis is mainly descriptive and stratified, but it does not adequately adjust for these confounders. The authors should perform multivariable analyses that include, at minimum, hypertension status, age, BMI, sex, region, parasitological status, and relevant clinical or lifestyle covariates if available. For beta diversity, PERMANOVA models should include these covariates, and dispersion should be tested. For differential abundance analysis, the design formula should include relevant covariates rather than only comparing groups descriptively.

 Without such analyses, statements attributing microbiota differences to hypertension should be substantially weakened.

 3. The statistical analysis is insufficiently described and partially inappropriate for the data structure

 The manuscript states that one-way ANOVA was used for diversity comparisons, but the rationale for this choice is not provided. Given the small and imbalanced subgroups, non-normal distributions, and multiple comparisons, non-parametric methods or model-based approaches may be more appropriate. The manuscript also presents several subgroup analyses by BMI, age, and region, but does not consistently provide p-values, effect sizes, confidence intervals, FDR-adjusted results, or sample sizes per subgroup.

 For beta diversity, ordination plots are interpreted visually, but formal statistical testing is not adequately reported. PERMANOVA results, R² values, p-values, and tests for homogeneity of dispersion should be included. For differential abundance, the authors mention DESeq2 and an FDR threshold, but no clear table of differentially abundant taxa with log2 fold changes, standard errors, adjusted p-values, and prevalence is provided.

 The authors should revise the statistical analysis section and present complete statistical outputs for alpha diversity, beta diversity, and differential abundance.

 4. Sample handling and storage may introduce bias

 The methods indicate that stool samples were collected by participants at home and delivered the following day, and that some samples were stored at -20°C while others were kept at room temperature for later analysis related to parasitic infections. This is a major issue for microbiome studies if not carefully controlled.

 The authors should clarify whether the samples used for 16S sequencing were all processed under the same storage conditions. If storage conditions differed among sequenced samples, this factor should be reported and included in the statistical analysis. If the room-temperature samples were not used for microbiome sequencing, this should be explicitly stated.

 5. Medication, diet, and lifestyle factors are insufficiently controlled

 The manuscript refers to diet and regional nutritional differences, but detailed dietary intake is not analyzed. Antihypertensive drugs, statins, proton pump inhibitors, metformin, laxatives, and other medications can affect the gut microbiota. Physical activity, alcohol intake, smoking, socioeconomic status, and stool consistency are also relevant covariates.

 The authors should report which medication and lifestyle data were collected and include them in the analysis where possible. If these data were not available, this limitation should be emphasized more strongly and conclusions should be moderated.

 6. Geographical origin as a potential confounder and effect modifier

 Given that participants were recruited from different regions of Peru, geographical origin should be explicitly considered in the analysis. In this context, region may capture differences in diet, altitude, urbanization, environmental exposures, parasitic burden, and sociocultural practices, all of which can strongly influence gut microbiota composition. Therefore, unadjusted comparisons between hypertensive and normotensive individuals may confound hypertension-related effects with region-associated microbial variation.

 The authors should adjust the main microbiome analyses for region or macroregion (coast, highlands, jungle), depending on sample size. For beta diversity, a multivariable PERMANOVA including hypertension status, age, BMI, sex, region/macroregion, parasitological status, and sample storage condition, if applicable, would be appropriate. Similar adjustment should be applied to alpha diversity and differential abundance analyses. A formal host genetic population structure analysis is only necessary if ancestry data are available or if ancestry-related claims are made.

 7. Conclusions and public health implications overreach the data

 The manuscript suggests “microbial signatures” of hypertension and “opportunities for preventive and therapeutic interventions.” These statements are premature given the cross-sectional design, small sample size, lack of multivariable adjustment, and 16S-based inference. The study can support exploratory associations and generate hypotheses, but it cannot establish causal microbial signatures or intervention targets.

 The conclusion should be rewritten to emphasize exploratory findings, regional heterogeneity, and the need for larger, longitudinal, controlled studies with shotgun metagenomics and metabolomics.

**Minor concerns**

1. The abstract states that hypertension affects around 19.4% of Peruvians over 15 years old, while the introduction reports 14.2%. This discrepancy should be corrected or explained.
2. The manuscript should consistently use “hypertension” rather than alternating between “high blood pressure” and “arterial hypertension,” unless a specific distinction is intended.
3. “16S ARNr” should be corrected to “16S rRNA.”
4. The manuscript should consistently use English terms in tables. For example, “Hipertensive,” “Si,” “Negativo,” “Sierra,” “Selva,” and “Costa” should be translated and corrected.
5. In Table 1, several p-values appear as “470,” “199,” “733,” etc. These likely correspond to 0.470, 0.199, and 0.733 and must be corrected.
6. Table 2“Yacht correction” should be corrected to “Yates correction.”
7. Table 2: “Cost” should be corrected to “Coast.”
8. “Trigerlcyids” or “Triglycerids” should be corrected to “Triglycerides.”
9. “Cuzco” and “Cusco” should be standardized.
10. Table 2 is very dense and difficult to read. It may be more appropriate as supplementary material.
11. Reference 4 appears unrelated to the specific claim about gut microbiota dysbiosis and hypertension. The authors should verify that all references support the statements they are attached to.
12. The manuscript would benefit from professional English editing. Several sentences are grammatically correct but unnatural or overly long.
13. The discussion contains repeated ideas, particularly around geography, diet, BMI, and age. It should be made more concise.
14. The authors should define all abbreviations at first use, including ASV, PCoA, NMDS, FDR, BMI, SCFA, and PICRUSt2.

Comments on figures and tables

 Table 1

 The table requires substantial formatting and language revision. P-values must be reported with decimals and appropriate precision. The authors should indicate which variables are presented as mean ± SD and which as median and IQR. The label “Adjusted blood pressure” is unclear and should probably be revised to “diastolic blood pressure” if that is what is intended.

 Table 2

 The table is difficult to interpret due to the number of columns and very small regional sample sizes. Several statistical comparisons may be unreliable because of sparse cells. The authors should consider moving this table to supplementary material and summarizing the key regional characteristics in the main text. Fisher’s exact test may be more appropriate than chi-square tests for sparse categorical data.

 Figure 1

 The heatmap is difficult to read. Genus labels and sample labels are too small. The authors should include annotation bars for hypertension status, region, age group, BMI category, and storage condition if applicable. It may be preferable to show only the top genera or provide the complete heatmap as supplementary material.

 Figures 2, 3, 6, 7, 10, and 11

 Stacked bar plots of mean relative abundance are descriptive but do not show variability across individuals. The authors should include sample sizes per group and consider complementary plots showing individual-level distributions. Claims of enrichment or absence should be supported by statistical testing.

 Figures 4, 8, and 12

 The boxplots should include sample sizes, statistical test results, and adjusted p-values where multiple comparisons are performed. If the differences are not statistically significant, the text and figure legends should avoid implying meaningful group differences.

Is the work clearly and accurately presented and does it cite the current literature?

Yes

If applicable, is the statistical analysis and its interpretation appropriate?

Partly

Are all the source data underlying the results available to ensure full reproducibility?

Partly

Is the study design appropriate and is the work technically sound?

Yes

Are the conclusions drawn adequately supported by the results?

Partly

Are sufficient details of methods and analysis provided to allow replication by others?

Partly

Reviewer Expertise:

Molecular oncology, bioinformatics, infectious diseases

I confirm that I have read this submission and believe that I have an appropriate level of expertise to confirm that it is of an acceptable scientific standard, however I have significant reservations, as outlined above.

# Reviewer report: Reviewer response for version 1

**DOI:** 10.5256/f1000research.188405.r491251

**Published:** 2026-06-19

**Authors:** Rosa Del campo Moreno

**Affiliations:** 1 Universidad de la Rioja, Logroño, La Rioja, Spain

In this study, the authors aimed to investigate the variability of the intestinal microbiota in subjects with and without hypertension from different regions of Peru. The main weakness of this article is that the causal relationship implied by the hypothesis is unclear. The text requires careful revision, as the authors have only identified an association between the microbiota composition of hypertensive subjects and normotensive controls.

 A critical point is whether the hypertensive subjects were receiving pharmacological treatment. If they were not, it would have been advisable to include an additional group of treated hypertensive patients. Moreover, hypertension is a heterogeneous condition; it would be recommended to stratify patients according to disease duration (years of evolution) and the quality of blood pressure control.

 The stratification by regional altitude is considered very interesting; however, it substantially reduces the sample size in each subgroup, thereby limiting the statistical power of the analyses.

 Although differences in parasite prevalence are mentioned, the specific parasite species identified are not reported. It should be recalled that parasites are also integral components of the gut microbiota.

 The figures have insufficient resolution and are largely illegible. Furthermore, some figures and tables still contain text in Spanish.

 I disagree with the use of functional prediction based solely on 16S rRNA gene sequencing (please ensure consistent and correct spelling, as it also appears in Spanish in some titles). Bacterial phylum names should not be italicized.

 The glucose values reported in Table 1 are particularly noteworthy, as hypertensive subjects exhibited lower glucose levels. Could this be explained by ongoing pharmacological treatment? A detailed description of the treatments received by the participants is essential. In addition, Table 1 contains Spanish words and shows inconsistent use of commas and periods as decimal separators. The unusually high p-values are also difficult to interpret.

 Finally, what is the scientific rationale for excluding subjects older than 70 years?

Is the work clearly and accurately presented and does it cite the current literature?

Partly

If applicable, is the statistical analysis and its interpretation appropriate?

I cannot comment. A qualified statistician is required.

Are all the source data underlying the results available to ensure full reproducibility?

Yes

Is the study design appropriate and is the work technically sound?

No

Are the conclusions drawn adequately supported by the results?

No

Are sufficient details of methods and analysis provided to allow replication by others?

Partly

Reviewer Expertise:

Microbiota and health

I confirm that I have read this submission and believe that I have an appropriate level of expertise to confirm that it is of an acceptable scientific standard, however I have significant reservations, as outlined above.
